# Genome-Wide Mapping of Decay Factor–mRNA Interactions in Yeast Identifies Nutrient-Responsive Transcripts as Targets of the Deadenylase Ccr4

**DOI:** 10.1534/g3.117.300415

**Published:** 2017-11-20

**Authors:** Jason E. Miller, Liye Zhang, Haoyang Jiang, Yunfei Li, B. Franklin Pugh, Joseph C. Reese

**Affiliations:** * Center for Eukaryotic Gene Regulation, Department of Biochemistry and Molecular Biology, The Pennsylvania State University, University Park, Pennsylvania 16802; † Center for RNA Molecular Biology, Department of Biochemistry and Molecular Biology, The Pennsylvania State University, University Park, Pennsylvania 16802; ‡ Center for Comparative Genomics and Bioinformatics, Department of Biochemistry and Molecular Biology, The Pennsylvania State University, University Park, Pennsylvania 16802

**Keywords:** Ccr4-Not, mRNA decay, Dhh1 RNA, immunoprecipitation-seq

## Abstract

The Ccr4 (carbon catabolite repression 4)-Not complex is a major regulator of stress responses that controls gene expression at multiple levels, from transcription to mRNA decay. Ccr4, a “core” subunit of the complex, is the main cytoplasmic deadenylase in *Saccharomyces cerevisiae*; however, its mRNA targets have not been mapped on a genome-wide scale. Here, we describe a genome-wide approach, RNA immunoprecipitation (RIP) high-throughput sequencing (RIP-seq), to identify the RNAs bound to Ccr4, and two proteins that associate with it, Dhh1 and Puf5. All three proteins were preferentially bound to lowly abundant mRNAs, most often at the 3′ end of the transcript. Furthermore, Ccr4, Dhh1, and Puf5 are recruited to mRNAs that are targeted by other RNA-binding proteins that promote decay and mRNA transport, and inhibit translation. Although Ccr4-Not regulates mRNA transcription and decay, Ccr4 recruitment to mRNAs correlates better with decay rates, suggesting it imparts greater control over transcript abundance through decay. Ccr4-enriched mRNAs are refractory to control by the other deadenylase complex in yeast, Pan2/3, suggesting a division of labor between these deadenylation complexes. Finally, Ccr4 and Dhh1 associate with mRNAs whose abundance increases during nutrient starvation, and those that fluctuate during metabolic and oxygen consumption cycles, which explains the known genetic connections between these factors and nutrient utilization and stress pathways.

An important aspect of gene regulation is the proper control of mRNA levels, which is the product of both synthesis and decay. Although the earliest studies placed the greatest attention on how mRNA levels are controlled through transcription, interest in the role of mRNA decay is increasing. In yeast, the canonical mode of degradation starts with the shortening of the poly(A) tail by Pan2/3p and the main cytoplasmic deadenylase, Ccr4p, which is part of the Ccr4-Not complex (Brown *et al.* 1996; Tucker *et al.* 2001; Chen *et al.* 2002; Goldstrohm and Wickens 2008; Wahle and Winkler 2013). Deadenylation is followed by the removal of the 5′ cap by Dcp1/2p, which leads to exonucleolytic cleavage of the mRNA in the 5′ to 3′ direction by Xrn1p (Eulalio *et al.* 2007). Alternatively, 3′ to 5′ decay is catalyzed by the exosome complex in a regulated manner (Schmid and Jensen 2008). Additional proteins bind specific regions on the mRNA, such as the 3′ UTR (untranslated region), to mediate decay and the localization of the mRNA (Goldstrohm and Wickens 2008). Remarkably, even among the most well-studied factors like Ccr4p, it remains unclear how and what regulates their recruitment to mRNAs across the transcriptome.

Ccr4-Not is composed of nine conserved core subunits that form a 0.9–1.2 MDa protein complex: Not1 through Not5, Caf1/Pop2, Ccr4, Caf40, and Caf130 (Miller and Reese 2012; Collart 2016). The Ccr4-Not complex interacts with a number of other proteins, such as the RNA-binding proteins (RBPs) Puf5 and the RNA helicase Dhh1 (Hata *et al.* 1998; Coller *et al.* 2001; Maillet and Collart 2002; Goldstrohm *et al.* 2006; Tarassov *et al.* 2008; Alhusaini and Coller 2016). Puf5 is a member of the Fem-binding family of proteins (Quenault *et al.* 2011) that binds to a motif in the 3′ UTR of mRNAs and enhances deadenylation by Ccr4-Not (Goldstrohm *et al.* 2006, 2007). Recent crystallography studies verified the interaction between Dhh1 and the N-terminus of Not1 (Chen *et al.* 2014; Mathys *et al.* 2014). Although Dhh1 binds to Ccr4-Not, it is much more abundant, and additionally inhibits translation and promotes the decapping of mRNAs (Ghaemmaghami *et al.* 2003; Coller and Parker 2005; Sweet *et al.* 2012; Radhakrishnan *et al.* 2016). Although these three proteins physically and genetically interact with each other, they play distinct functions in mRNA regulation (Miller and Reese 2012; Presnyak and Coller 2013); thus, it is unclear to what extent their mRNA targets overlap.

One of the features of Ccr4-Not that makes it an intriguing complex to study is that it regulates both transcription and mRNA degradation (Miller and Reese 2012; Collart 2016). The deletion of Ccr4-Not subunits affects the abundance, decay, and synthesis of many mRNAs (Grigull *et al.* 2004; Cui *et al.* 2008; Molina-Navarro *et al.* 2008; Sun *et al.* 2012, 2013). However, the interpretation of gene expression changes in deletion mutants, while valuable, can be influenced by complex genetic interactions and secondary effects. In the aforementioned studies, it remains unknown which changes in mRNA expression are direct and which result from perturbed mRNA synthesis or decay. Therefore, identifying the direct mRNA targets of Ccr4-Not could shed light on the molecular underpinnings behind how RNA abundance, decay, and synthesis are controlled across the transcriptome.

Here, a modified RIP-seq procedure was used to identify transcripts associated with Ccr4, Dhh1, and Puf5. In addition to showing that these factors are recruited to many of the same mRNAs, the analysis of the mRNA targets suggests that Ccr4 imparts its greatest influence on gene regulation at the level of decay *vs.* synthesis. Our study has illuminated the interplay between the two cytoplasmic deadenylases in mRNA decay. Additionally, the recruitment of these three factors negatively correlated with ribosome density, suggesting new insights into the relationship between mRNA decay and translation. Finally, we show that Ccr4 and Dhh1 bind mRNAs that fluctuate during the yeast metabolic cycle (YMC), suggesting a role for post-transcriptional regulation in reshaping the transcriptome in response to changing environmental conditions.

## Materials and Methods

### Strain construction

All strains were constructed in the BY4741 background using published protocols (Longtine *et al.* 1998). A list of strains is contained in Supplemental Material, Table S5 in File S5.

### RIP

RIP-seq was based upon a previous version of the procedure (Dutta *et al.* 2011), but incorporating changes for high-throughput sequencing of mRNAs. Cells were grown in 1 L of YPAD (2% peptone, 1% yeast extract, 0.02 mg/ml adenine sulfate, and 2% dextrose) at 30° to an OD_600_ ∼0.9, and then formaldehyde was added to 1% (v/v) for 15 min. Cross-linking was quenched with glycine (final = 136 mM) for 5 min. Subsequent steps used buffers prepared with diethyl polycarbonate-treated (DEPC) water. Cells were harvested and washed in ice-cold STE (10 mM Tris-HCl pH 8, 1 mM EDTA, 50 mM NaCl, 0.5 mM PMSF, and 1 mM benzamidine-HCl) and frozen at −80°. Cells were resuspended in FA-lysis buffer (50 mM HEPES/KOH pH 7.5, 150 mM NaCl, 1% Triton X-100, and 0.1% sodium deoxycholate) containing protease inhibitors (2 μg/ml leupeptin, 3 μg/ml aprotinin, 2 μg/ml pepstatin A, 1 μg/ml chymostatin, 1 mM benzamidine-HCl, and 0.5 mM PMSF). Cells were disrupted by vortexing in the presence of glass beads for 45 min at 4°, and then 200 µl FA-lysis buffer was added to each tube and mixed for 30 sec, twice. Cell lysate was then transferred to two 15 ml polystyrene tubes (∼1.8 ml lysate in each), sonicated with two 30 sec pulses using a Bioruptor sonicator (Diagenode, Philadelphia, PA), and then clarified by centrifugation twice at 4° at 14,000 rpm. Protein content was measured by Bradford assay (Bio-Rad) using BSA as a standard. Samples with > 7 mg/ml protein concentration were used in RIP.

Whole-cell extract (2.5 ml) was diluted with an equal volume of FA-lysis buffer (containing protease inhibitors). MgCl_2_ and CaCl_2_ were added to concentrations of 25 and 5 mM, respectively, and then RNase-free DNase I (Worthington, Lakewood, NJ) was added to 174 units per ml. The samples were incubated for 90 min at 30°. EDTA was added to 50 mM and the samples were placed on ice. Samples were spun for 10 min at 14,000 rpm at 4°, and then 1 ml of supernatant was transferred to four tubes containing 15 µl of protein A–sepharose (GE Healthcare) slurry containing 3.5 µl 9E10 (anti-myc) monoclonal antibody from ascites fluid (Biolegend). In experiments where RT-qPCR of RNA was used for validation, Protein A beads bound with 7 µl of purified 9E10 antibody were used. The samples were incubated overnight at 4° with rotation. Beads were washed three times with FA-lysis buffer, two times with FA-wash buffer 2 (50 mM HEPES/KOH pH 7.5, 1 mM EDTA, 1% Triton X-100, 0.1% sodium deoxycholate, 0.5 M NaCl, 0.5 M PMSF, and 1 mM benzamidine-HCl), three times with FA-wash buffer 3 (0.25 M LiCl, 1% NP-40, 1% sodium deoxycholate, 1 mM EDTA, 10 mM Tris-HCl pH 8.0, 0.5 M PMSF, and 1 mM benzamidine-HCl), and two times with TE buffer (10 mM Tris-HCl and 1 mM EDTA, pH 8). RNA was eluted off the beads at 65° for 20 min in 450 µl elution buffer [25 mM Tris-HCl (pH 7.5), 1 mM EDTA, 0.2 M NaCl, and 0.5% SDS]. To reverse cross-links, proteinase K was added to 70 μg/ml and incubated at 65° for 5 hr. Input samples (100 µl of extract) were supplemented with 350 μl elution buffer, 10 μl 10% SDS, and then incubated at 65° for 5 hr. The RNA was purified using an acid–phenol (pH 4.8)/chloroform (1:1) extraction followed by ethanol precipitation in the presence of 20 μg glycogen. Samples were resuspended in DEPC water, and then treated with DNase once again. RNA was extracted with acid–phenol (pH 4.8)/chloroform/isoamyl-alcohol (25:24:1) and ethanol-precipitated in the presence of 10 μg glycogen. The pellet was resuspended in DEPC water and the nucleic acids quantified using a Nanodrop instrument.

### Library construction, sequencing, and read mapping

Each sample was performed in biological triplicate except for the input, which was performed in duplicate. First, 0.5 μg of RNA from each replicate was incubated with RNase III (Ambion cat#2290) for 10 min at 37° to fragment the RNA and prepare the ends for linker addition. RNA was then concentrated using a concentration module (Invitrogen). The size and quality of the RNA was examined on a 2100 Agilent Bioanalyzer. cDNA libraries were then prepared using the SOLiD Total RNA-Seq Kit (PN 4445374). In brief, adapters were ligated onto the fragmented RNA and reverse transcribed using the SOLiD Total RNA kit. After two rounds of purification and size selection using the Agencourt AMPure XP Reagent, the library was PCR amplified and then purified using the Invitrogen PureLink PCR Micro Kit. Yield and size of amplified DNA was assessed using a 2100 Agilent Bioanalyzer. Each amplified DNA library was then clonally amplified in an emulsion PCR reaction. RIP and total RNA samples were sequenced on a SOLiD 4 platform.

All samples were downloaded separately by barcode and reads were mapped to the 2007 *Saccharomyces cerevisiae* reference genome (sg7) using SHRiMP version 2.2.2 (Rumble *et al.* 2009). Reads were trimmed 15 bp from the 3′ end as a quality control measure. During mapping, SHRiMP2 calculated a score for each read based upon mismatches, and reads with < 90% of the maximum possible score were filtered out (a 90% threshold is similar to allowing for three mismatches).

### Composite plots

Python scripts were used to create composite plots. The purpose of the scripts can be conceptually described as follows. First, the reads were aligned to a TSS (Transcription Start Site) or a TTS (Transcription Termination Site) from a published source (David *et al.* 2006), in a strand-specific manner. The read density was summarized around TSS or TTS mRNAs, then corrected for differences in total uniquely mappable reads so that the final output would be average read density per 100 million reads. Finally, the data were binned into 15 bp bins and smoothed using a 6 bp sliding window average.

### Enrichment calculation

See Figure S1 in File S5 for a diagram of the enrichment calculation. The reads per gene were tabulated after aligning reads to TSSs and TTSs identified from a published source (David *et al.* 2006). Counts per gene were used to measure reproducibility. The abundant reads from ribosomal RNAs were filtered out prior to measuring the reproducibility between the samples. To calculate enrichment, two of three replicates were processed at a time in all three combinations (*i.e.*, rep 1 *vs.* rep 2, rep 2 *vs.* rep 3, and rep 1 *vs.* rep 3) then averaged if the false discovery rate (FDR) was < 1%. Enrichment was calculated in edgeR for each gene by dividing reads in the immunoprecipitation (IP) sample by reads in the mock IP, while controlling for differences in library size among samples, (Robinson *et al.* 2010). One count was added to all genes using the prior.count argument in order to avoid infinite log_2_ values resulting from 0 reads in the mock IP. Log_2_ and p-values were calculated using the edgeR exacTest (assuming the Poisson model) and the Benjamini–Hochberg method was used to correct p-values for multiple testing (Benjamini and Hochberg 1995). To identify the regions of the mRNA bound by each factor, enrichment within the 5′ UTR, coding region, and 3′ UTR, reads were counted in a strand-specific manner using BEDOPS (Neph *et al.* 2012) and published gene annotations (Nagalakshmi *et al.* 2008). Following this step, enrichment was calculated in the same manner as for the whole RNA, as described in Figure S1 in File S5. Enrichment was also calculated across the length of mRNAs based on gene length as follows. Each replicate was first split into reads that aligned to the first-third, middle-third, and last-third of each gene before calculating enrichment. Following this step, enrichment was calculated in the same manner as for the whole RNA. For intronic analysis, HOMER was used to count reads per gene or per intron (Heinz *et al.* 2010). Enrichment was calculated separately for intronic regions using the pipeline described in Figure S1 in File S5, with the exception of using the saccer2 reference genome. A twofold enrichment cutoff was used to select for enriched intronic regions.

### Gene ontology (GO) terms analysis

For GO terms analysis, the percent ranks for enrichment values were first calculated using the =PERCENTRANK() function in Microsoft Excel. The genes among the top 20th percentile of enrichment values along with a list of all genes (“background population”) were submitted to an online GO term finder (go.princeton.edu; Boyle *et al.* 2004), after which p-values were corrected using the Bonferroni method. The following terms were not included in the figures representing the GO terms output due to their redundant nature: “regulation of cellular process,” “regulation of biological process,” “biological regulation,” and “biological_process.” The 232 mRNAs connected to “organelle organization” in the Dhh1 GO Term Finder output were submitted to GO Slim Mapper using default settings (http://www.yeastgenome.org/cgi-bin/GO/goSlimMapper.pl).

### Motif analysis

Sequence motifs were identified using FIRE (finding informative regulatory elements), which was performed either locally or on the iGET website (https://iget.c2b2.columbia.edu) using default settings to analyze “discrete” or “continuous” data on Puf5, Dhh1, and Ccr4 unstressed enrichment values using 6, 7, and 8 bins, respectively (Elemento *et al.* 2007).

### Statistical and visual analysis

Correlations were calculated in R using the cor, cor.test, or corr.test (from the “psych” package) functions. Heatscatter graphs were made using the R package “LSD.” Circles and ellipses for Venn Diagrams were made using the program eulerAPE (Micallef and Rodgers 2014) or using the package “VennDiagram” in R. Other graphs were generated using base R graphics or in Microsoft Excel. Screen shots were generated in IGV after adjusting tag count for differences in library size (Robinson *et al.* 2011; Thorvaldsdottir *et al.* 2013). Average reads per kilobase of transcript per million reads mapped (RPKM) was calculated by first adding 0.5 to each gene so as to not eventually take the log of zero, then calculating RPKM for each input separately, and finally taking the average of the two inputs.

#### External data sources:

The Comparative Dynamic Transcriptome Analysis (cDTA) data (Sun *et al.* 2012, 2013) can be downloaded from the Cramer laboratory website http://www.cramer.genzentrum.lmu.de/movies/, or can be found in the supplemental information associated with the respective publications. The RIP-Chip (Gerber *et al.* 2004; Hogan *et al.* 2008), Ribo-seq (Gerashchenko *et al.* 2012), Dhh1 CLIP-seq (Mitchell *et al.* 2013), Not1 RIP-seq (Gupta *et al.* 2016), gene run-on (GRO-Chip) (Molina-Navarro *et al.* 2008), and genome-wide ChIP (Venters *et al.* 2011) data sets were downloaded from their respective online supplemental files associated with the publication. Gene expression array data (Bradley *et al.* 2009; Arribere *et al.* 2011) was also found in the online supplemental files associated with their respective publication. The Gasch *et al.* (2000) microarray data were located online at http://downloads.yeastgenome.org/published_datasets/Expression_connection_data/. YMC gene expression values can be found at http://moment.utmb.edu/cgi-bin/dload.cgi, while processed data were obtained via E-mail (see *Acknowledgments*).

#### RT-qPCR verification:

RNA was isolated from biological triplicate (cells were grown overnight in separate flasks before harvesting) samples. Equivalent amounts of RNA from input and IP samples (∼350 ng) were used to produce cDNA using random hexadeoxynucleotide primers ([final] = 0.01 μg/μl, Promega #C1181) and AMV reverse transcriptase (0.4 unit/µl) for 1 hr at 42° in a 15 μl volume. Real-time PCR was performed using Quanta PerfeCTa SYBR Green SuperMix in a 96-well plate (in technical triplicate) in an Applied Biosystems StepOnePlus machine. For each input and IP, the average Ct value was calculated from the technical triplicates. Then the %IP was calculated using the equation 2^(Ct_Input(avg)_−Ct_IP(avg)_). The average and SD of three biological replicates was reported using the AVERAGE and STDEV functions in Microsoft Excel. See File S5 for a list of primers.

### Data availability

The data from this study will be deposited with the Gene Expression Omnibus, accession no. GSE72366.

## Results

### Factors involved in different aspects of mRNA decay specifically target the 3′ UTR of the same collection of mRNAs

RNA CLIP procedures are often used to map RNA–protein interactions, but the harvesting, processing, and UV cross-linking steps can introduce stress and change the RNA–protein interaction landscape (Mitchell *et al.* 2013; Riley and Steitz 2013). We used formaldehyde (FA) cross-linking of cells in culture, which rapidly traps protein–nucleic acid interactions in their physiological state with minimal-to-no stress response. All three proteins under study here contact RNA directly (Chen *et al.* 2002; Cheng *et al.* 2005; Goldstrohm *et al.* 2006; Dutta *et al.* 2011), but FA can potentially cross-link a protein to RNA indirectly via another protein that is in direct contact with the RNA. Percentages of cross-linking efficiency are protein-dependent and hard to estimate *in vivo*, but it is widely accepted that a protein is cross-linked to a nucleic acid only a fraction of the time; therefore, “double-hit” cross-linking is expected to occur at a low frequency. Furthermore, even if the recruitment of a protein to an RNA is through a bridging protein, this in itself is desirable and informative because it indicates that it could regulate the fate of that RNA. Finally, RNA isolation and library construction used in this procedure do not target the poly(A) tail; therefore, potential technical artifacts caused by variations in the poly(A) tail length of messages under the control of the decay machinery are reduced.

We identified RNAs associated with Ccr4, Dhh1, and Puf5 as representatives of the mRNA decay machinery because they play different roles in the regulation of mRNAs. Ccr4 is the major mRNA deadenylase; thus, its binding can estimate the decay potential of transcripts in the same way that the binding of RNA polymerase II to a gene is used to determine its transcription level. Ccr4 was chosen for study, as opposed to Pan2/3, because Ccr4 plays a more prominent role in regulating poly(A) tail length and degradation rates (Tucker *et al.* 2001; Sun *et al.* 2013). Until this study, the recruitment of Ccr4 to mRNAs has not been reported on a transcriptome-wide scale in *S. cerevisiae*.

**Figure 1:**
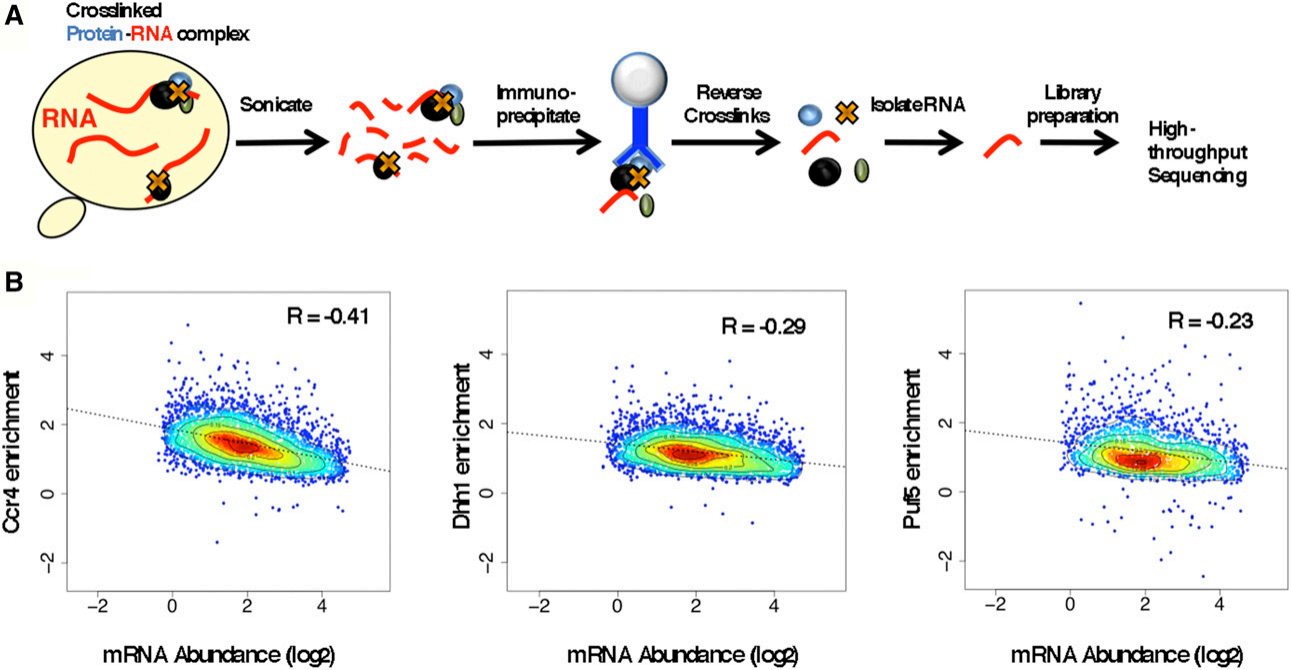
Identification of Ccr4, Dhh1, and Puf5 RNA targets. (A) An illustration of the RNA immunoprecipitation high-throughput sequencing (RIP-seq) procedure (see *Materials and Methods* for details). (B) Heat scatter plots of RIP-seq enrichment values correlated with mRNA abundance levels (mRNA/cell) on a log_2_ scale [measured in Sun *et al.* (2012)]. The *R* values within the graphs represent Pearson correlation values. The color refers to the relative concentration of data points in a single area, *i.e.*, red refers to greater density and blue to lower density. The dotted black trend line represents the least squares regression.

RNAs isolated from the immunoprecipitates of extracts from cells containing a myc epitope-tagged version of each protein (*e.g.*, Ccr4 IP), and from extracts of untagged cells (from here on referred to as UT IP), were identified by high-throughput sequencing (Figure 1A). The assays were carried out in triplicate and the reproducibility among all samples was very high (*R*^2^ values > 0.93) (Table S1 in File S5 and File S1). Enrichment was calculated using the workflow described in the *Materials and Methods* and as illustrated in Figure S1 in File S5. The vast majority (> 95%) of the transcripts enriched by each protein were mRNAs (Figure S2 in File S5, File S6). We compared the enrichment values of each decay factor to mRNA abundance (Sun *et al.* 2012) to address the question of whether these proteins are recruited to mRNAs (targeted) or if the enrichment is simply a reflection of their relative abundance in the cell (nonspecific). The mRNA enrichment to all three proteins displayed a negative correlation with abundance (Figure 1B), with the Ccr4 data showing the strongest anticorrelation (−0.41 Pearson correlation). The negative correlation between RIP-seq enrichment and abundance was observed using the input RNA-seq samples from this study and other published measurements of mRNA abundance (Table S2 in File S5). Thus, these observations are robust and applicable to multiple data sets. Enrichment of a transcript did not correlate with its length on a global scale either (Table S3 in File S5). Thus, the data suggest that these proteins are recruited to specific mRNAs, rather than associating with them indiscriminately based on abundance or length.

**Figure 2:**
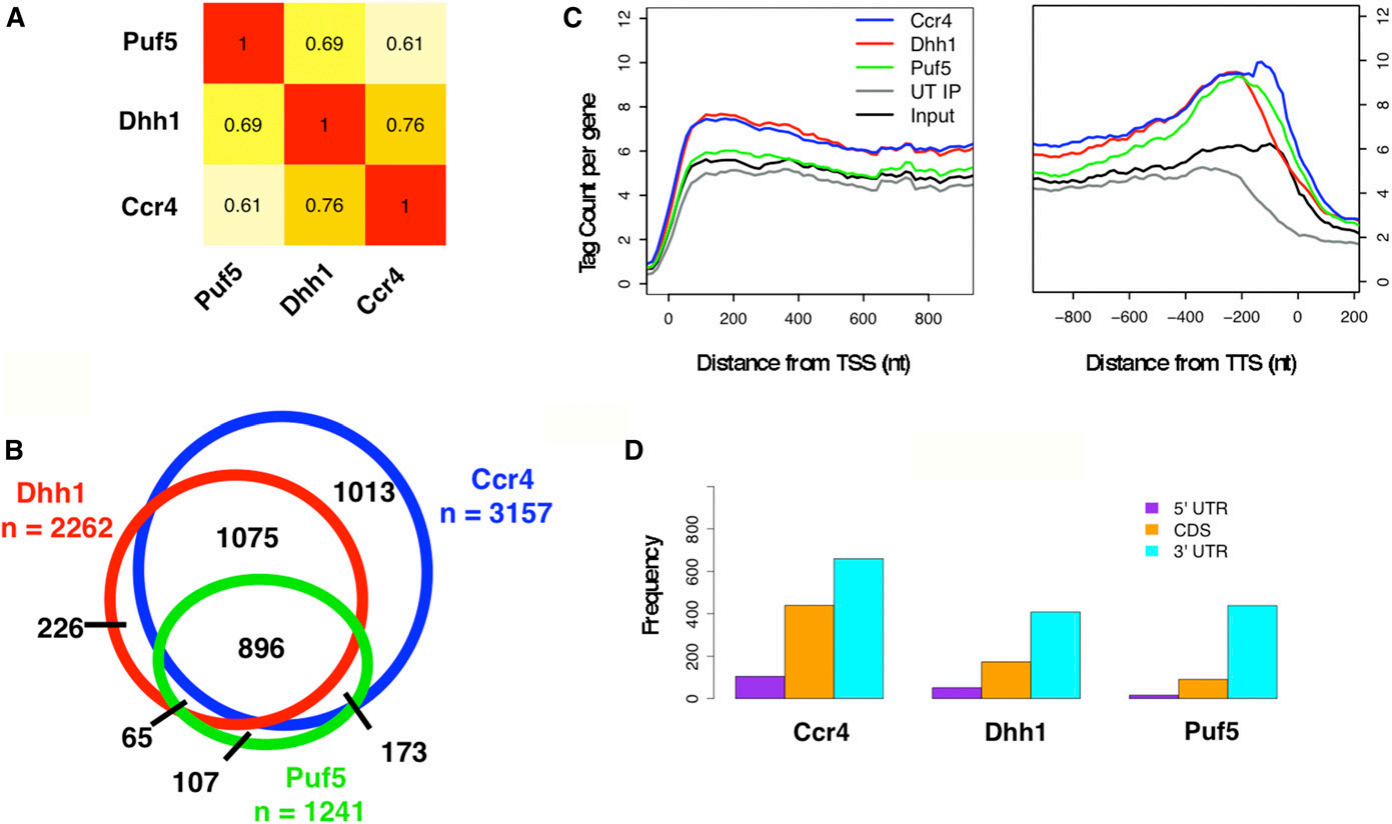
RNA immunoprecipitation high-throughput sequencing (RIP-seq) reveals that Ccr4, Dhh1, and Puf5 target the same collection of mRNAs (A). Pearson correlations of pairwise RIP-seq sample enrichment values. (B) Venn diagram of targets of Puf5 (green), Dhh1 (red), and Ccr4 (blue) with enrichment values > 1 log_2_ in two of the three biological replicates using a false discover rate < 1%. (C) Composite plots of mRNA sequencing read density of all mRNAs with a length of > 400 nucleotides (nt) (*n* = 4565). The reads were aligned relative to the TSS [transcription Start Site (left)] and TTS [Transcript Termination Site (Right)] in nt. The TSS and TTS were identified from another study (David *et al.* 2006). Tag count per gene was normalized to 100 million reads to account for differences in library sizes. (D) The enrichment of each factor was calculated over the 5′ untranslated region (UTR), coding sequence (CDS), and 3′ UTR of mRNAs. The number of mRNAs (> 400 nt) with more than fourfold enrichment is displayed on the *y*-axis.

Ccr4, Dhh1, and Puf5 display physical and genetic interactions with each other; however, they play different roles in mRNA decay. For example, Ccr4 deadenylates the poly(A) tail located at the 3′ end, and Dhh1 regulates translation and decapping occurring at the 5′ end of mRNAs (Tucker *et al.* 2001; Fischer and Weis 2002; Coller and Parker 2005). Furthermore, Dhh1 is 15 and 34 times more abundant than either Ccr4 or Puf5, respectively (Ghaemmaghami *et al.* 2003); thus, it was unclear how well the targets of these proteins would overlap and if they would be found at the same location on mRNAs. A pairwise comparison of the enrichment values of RNAs bound to each protein revealed a high level of correlation (*R* > 0.6, Figure 2A and Figure S3, A–C in File S5). Additionally, we validated several targets using an RT-PCR approach, which revealed a good-to-strong correlation between RIP-seq values and those obtained by RT-PCR (Figure S4, A and B in File S5).

Next, we examined the overlap of RNA targets of the three factors with each other. For this purpose, we identified RNAs that were enriched at least twofold (> 1 log_2_) with an FDR < 1% and present in at least two of the three replicates. Using these criteria, 3157, 2262, and 1241 RNAs were found to be associated with Ccr4, Dhh1, and Puf5, respectively (Figure 2B). There was a strong overlap among the targets of these three proteins. Interestingly, most mRNAs bound by Dhh1 or Puf5 were also targets of Ccr4 (Figure 2B). These results suggest that the physical interactions between these proteins that have been identified in previous studies reflect their coregulation of mRNAs *in vivo*. Furthermore, the overlap of Ccr4 and Puf5 targets is consistent with studies that have shown that Puf5 recruits the Ccr4-Not complex to the 3′ UTR of the *HO* mRNA (Goldstrohm *et al.* 2006, 2007). Moreover, motifs (HUGUANHAD) with likeness (underlined portion is similar) to both Puf5 (UGUAAYAWUA) and the related RBP Puf4 (WHUGUAHAWUA) were overrepresented among the RNAs associated with all three proteins (p-value < 10^−4^, Figure S3D in File S5) (Gerber *et al.* 2004). The discovery of motifs similar to both Puf4- and Puf5-binding sites in the enriched mRNAs may be explained by the ability of Puf5 to adapt to variations in the RNA motif and recognize related sequences (Wilinski *et al.* 2015). Although Ccr4 was detected with the vast majority of mRNAs associated with Dhh1 and/or Puf5, an additional ∼1000 mRNAs were exclusive to the Ccr4 data set. These messages were depleted of the Puf5 and/or Puf4 motifs (Figure 2B and Figure S3D in File S5, p-value < 10^−5^), and Ccr4 is likely recruited to this group of mRNAs by other RBPs (see below).

The 5′ and 3′ UTRs of mRNAs contain sequences that control the translation, localization, and decay of mRNAs (Keene 2007; Hogan *et al.* 2008; Lee and Lykke-Andersen 2013). RIP sample preparation required a sonication step to solubilize cross-linked RNA–protein complexes, resulting in the shearing of RNAs to ∼200–600 nucleotides (nt). Thus, information on the spatial location of these proteins on mRNAs can be obtained. Composite plots were generated by aligning the sequencing reads to the TSS and the transcript TTS identified from a published high-resolution mapping of the transcriptome (David *et al.* 2006). A strong enrichment of Puf5 was observed over the 3′ end of mRNAs (Figure 2C), which agrees with studies showing that Puf5 binds to the 3′ UTR of mRNAs (Gerber *et al.* 2004; Goldstrohm *et al.* 2006). Where Ccr4 binds on an mRNA has not been examined on a global scale, but its function in deadenylation predicts that it would bind near the poly(A) tail. The mapping of reads from the Ccr4 IP revealed that it is preferentially recruited to 3′ ends of mRNAs (Figure 2C). Likewise, Dhh1 showed a stronger enrichment to the 3′ end of mRNAs. While the cross-linking of Ccr4 and Dhh1 was strongest at the 3′ end of mRNAs, reads above background were also observed in the middle and 5′ end. The detection of Ccr4 and Dhh1 in the body of the mRNA is unlikely to result from incompletely fractionated mRNAs during sample preparation, because all three samples were processed identically and equivalent enrichment of Puf5 along the length of the mRNA was not observed. While it was unexpected that Ccr4 would be enriched at both ends of mRNAs, our results with Dhh1 agree with a CLIP-seq study conducted in stressed cells, which observed it cross-linking to both the 5′ and 3′ ends of transcripts (Mitchell *et al.* 2013). The cross-linking of Ccr4 and Dhh1 at multiple locations on the transcript could result from the packaging of mRNAs into mRNPs, and the juxtaposition of the 5′ and 3′ ends of the RNA caused by associations between factors binding to the 5′ and 3′ ends. Indeed, Dhh1 interacts with both Ccr4-Not and factors associated with the 5′ cap of mRNAs (Hata *et al.* 1998; Maillet and Collart 2002; Nissan *et al.* 2010).

Composite plots can be biased by a large number of reads from a few mRNAs or can underestimate the extent of recruitment to a smaller number of transcripts. Therefore, we calculated the enrichment of each factor over the 5′ UTR, the coding sequence (CDS), and the 3′ UTR of mRNAs with a length > 400 nt. The frequency (number of mRNAs) that showed strong enrichment over these regions was calculated and displayed in Figure 2D. Similar to what was observed in the composite plots, the results indicate that each protein was associated predominantly with the 3′ UTR of mRNAs, and that Dhh1 and Ccr4 were detected at the 5′ UTR and CDS of more mRNAs than Puf5. An alternative way to analyze this is to separate the mRNAs into thirds, based on lengths, and calculate the number of RNAs where enrichment was detected at the 5′ ends, middle, or 3′ ends of the mRNA. Here too, the results indicate that each protein predominantly associated with the 3′ ends of the mRNA (Figure S5 in File S3). The recruitment of Puf5, Dhh1, and Ccr4 to the 3′ ends of mRNAs was observed at individual mRNAs (Figure S6 in File S5).

This is the first study to map mRNAs bound by Ccr4; however, targets of Puf5 and Dhh1 have been identified using RIP-Chip and CLIP-seq methods. We next compared our Dhh1 and Puf5 targets to published data sets. A native RIP-CHIP procedure conducted on Puf5 identified far fewer RNA targets than we did (only 224; Gerber *et al.* 2004), which may be explained by a lack of a cross-linking step and/or a high background of native RIP procedures. Nonetheless, we identified many of the same targets, and a significant positive correlation was observed between the RIP-Chip study and our own data (Figure S7, A and B in File S5).

The number of transcripts bound to Dhh1 in our study differs significantly from two other studies that used native (no cross-linking) conditions. When native RIP-Chip and RIP-seq methods were employed, < 80 Dhh1-enriched RNAs were detected (Hogan *et al.* 2008; Cary *et al.* 2015). Considering the high abundance of Dhh1 and its known functions in mRNA regulation, one can surmise that omitting a cross-linking step greatly underestimated the number of targets. Dhh1 targets were identified using CLIP-seq in cells undergoing glucose deprivation stress (Mitchell *et al.* 2013). This study identified 299 high- and 1838 low-confidence mRNAs (twofold cutoff and 2% FDR). A comparison of the targets identified in our study (2262 targets) to those identified in the CLIP-seq study (1838 targets) found relatively little overlap between the two data sets (548 targets; Figure S7C in File S5). Since the CLIP-seq study was conducted in cells deprived of a carbon source (stressed), and ours was carried out in rich media, the relatively weak overlap between the two studies is not surprising, considering that glucose starvation causes strong processing body (p-body) formation and presumably widespread remodeling of RNA–protein interactions (Teixeira *et al.* 2005; Mitchell *et al.* 2013).

### The mRNA decay machinery shares a regulatory network with other RBPs

**Figure 3:**
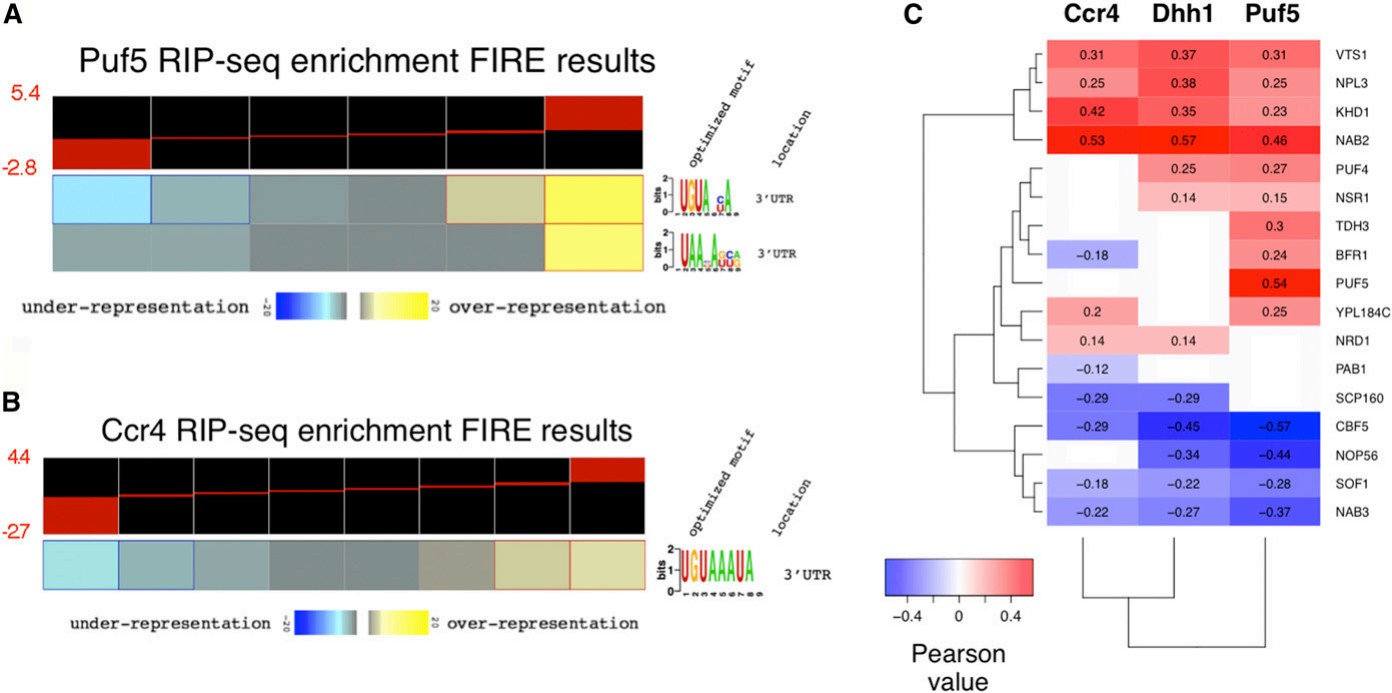
The decay machinery is directed to mRNAs through a network of RNA-binding proteins (RBPs). Enrichment values were submitted to FIRE (finding informative regulatory elements; Elemento *et al.* 2007) using default setting options for Puf5 (A) and Ccr4 (B). Values were binned into six and eight bins, respectively. The black and red heatmap represents the distribution of enrichment values contained within each bin (see range of enrichment values to the left in red font). Yellow bins represent groups of genes that have a significant overrepresentation of the motif, while blue represent bins where the motif is significantly underrepresented. See Elemento *et al.* (2007) for a detailed description of how the optimized motifs and location were attained. (C) Pearson correlations between RNA immunoprecipitation high-throughput sequencing (RIP-seq) enrichment values and enrichment values of multiple RBP targets from another study (Hogan *et al.* 2008). Multiple testing correction was performed and only correlations that met a false discover rate < 1% threshold are included.

Decay factors are recruited to mRNAs by RBPs that recognize sequence motifs located within the 3′ UTR (Goldstrohm and Wickens 2008; Quenault *et al.* 2011). Information on how Ccr4 and Dhh1 are recruited to mRNAs can be obtained by identifying sequence motifs in the bound mRNAs and by identifying overlap with publicly available RNA–protein interaction data sets. A search for sequence motifs among our highly enriched Puf5 targets using FIRE (Elemento *et al.* 2007) identified a motif resembling the Puf5 motif, as defined by the FIRE program (Figure 3A, p-value = 1.3 × 10^−12^). Identification of the Puf5 motif in our data set, and the correlation between our targets and those identified in other studies (Figure 3A and Figure S7, A and B in File S5), validate our RIP-seq procedure. Ccr4-Not is not known to display sequence-specific RNA binding, but instead is recruited to mRNAs via RBPs. The overlap of mRNA targets of Ccr4 and Puf5 suggests that Puf5 is a candidate (Figure 2, A and B), and we also discovered that the binding motif for Puf3 was overrepresented in the most highly enriched Ccr4 targets (p-value = 6.5 × 10^−4^, Figure 3B). It should be noted that the Puf3 motif emerging from the FIRE analysis of our data is a more restricted version than the motif identified in another study (Gerber *et al.* 2004). Finding the Puf3 motif overrepresented in Ccr4 targets is in good agreement with a study suggesting that Puf3 interacts with Ccr4 to regulate deadenylation of *COX17* mRNA (Lee *et al.* 2010). This, and our own data, suggests that Puf3 plays a significant role in recruiting Ccr4 to mRNAs across the genome. Neither our nor the published CLIP-seq study identified a motif significantly enriched in Dhh1-bound transcripts (Mitchell *et al.* 2013).

To find other RBPs that coregulate targets of Ccr4, Dhh1, and Puf5, correlations were calculated between the enrichment values from this study and those of other RIP-Chip studies (Hogan *et al.* 2008) (Figure 3C). Generally, Ccr4, Dhh1, and Puf5 enrichment positively correlated with targets of sequence-specific RBPs that have been implicated in mRNA decay or translational repression, including Vts1, Puf4, and Khd1. Ccr4-Not, Dhh1, and Puf5 have genetic and physical interactions with Puf4 and Vts1 (Hook *et al.* 2007; Rendl *et al.* 2008; Tarassov *et al.* 2008), and Ccr4 and Khd1 regulate the mRNAs of *LRG1* and *ROM2* [also identified in this work (File S2)] (Ito *et al.* 2011). Furthermore, there was a strong correlation between the mRNA targets identified here and those bound to Nab2 and Npl3, two nuclear poly(A)-binding proteins connected to mRNA export (Wilson *et al.* 1994; Lee *et al.* 1996; Hector *et al.* 2002; Marfatia *et al.* 2003). Physical interactions between Nab2 and Ccr4-Not complexes have been identified, suggesting that Ccr4-Not regulates some aspect of mRNA transport (Kerr *et al.* 2011). Nab2 binds poly(A) tails, and while thought to protect mRNAs from degradation, may aid in the recruitment of Ccr4-Not to mRNAs in the nucleus. In contrast, enrichment of the decay factors examined here negatively correlated with the targets of factors involved in ribosomal biogenesis (*i.e*., Cbf5, Nop56, and Sof1).

### Ccr4-Not directly regulates both mRNA synthesis and decay, but identification of its targets suggests that its role in decay predominates in gene expression control

The function of a gene is often inferred from gene expression profiling of mutants. However, interpretation of this type of data is difficult if the gene of interest has multiple functions in the cell. For example, Ccr4 and Dhh1 associate with RNA polymerase II and regulate transcription at the initiation and elongation stages of mRNA synthesis, and also function in decay (Deluen *et al.* 2002; Kruk *et al.* 2011; Miller and Reese 2012). Deleting Ccr4-Not subunits result in both increased and decreased expression of many genes (Cui *et al.* 2008; Azzouz *et al.* 2009), but which effects result from changes in transcription *vs.* decay are not known.

**Figure 4:**
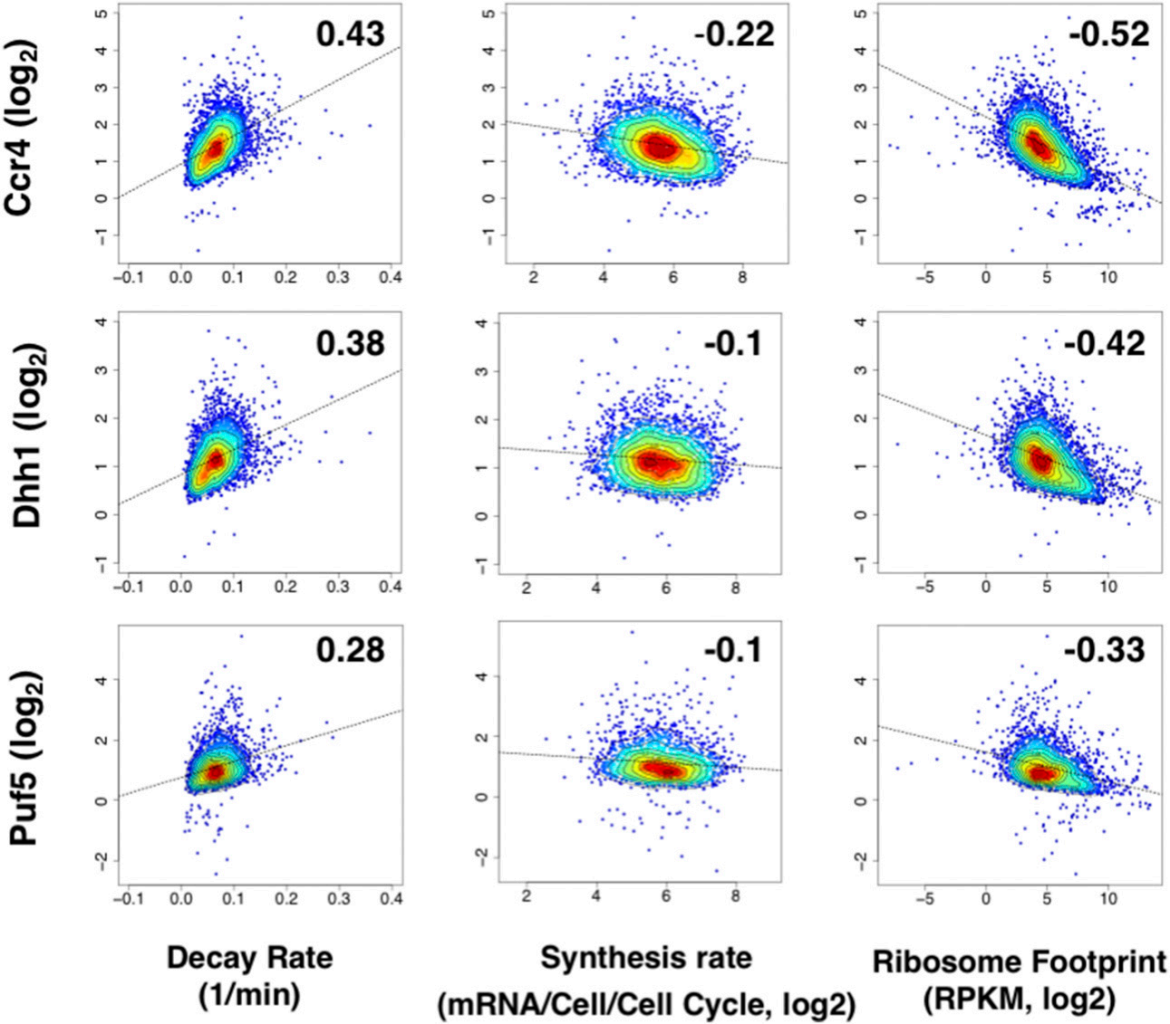
Recruitment of Ccr4, Dhh1, and Puf5 correlates with decay and not synthesis rates. Pair-wise comparisons of enrichment values (*y*-axis) *vs.* decay rates (1/min) and synthesis rates (mRNA/Cell/Cell Cycle, Log_2_) from Sun *et al.* (2012) and ribosome footprints [reads per kilobase of transcript per million reads mapped (RPKM), Log_2_] from Gerashchenko *et al.* (2012). Numbers within the graphs represent Pearson correlation values. The dotted black trend line represents the least squares line of regression.

RIP-seq enrichment data were compared to the decay and synthesis rates of mRNAs determined by cDTA, which measures these parameters with minimal perturbations by metabolic labeling of mRNAs (Sun *et al.* 2012). Ccr4 and Dhh1 enrichment showed a positive correlation with mRNA decay rates (*R* = 0.43 and 0.38, respectively) (Figure 4), which is consistent with their role in mRNA decay (Tucker *et al.* 2001, 2002). A positive correlation with decay rates, albeit not as strong, was observed also for Puf5-enriched mRNAs (*R* = 0.28). When the correlation between enrichment and decay rate was calculated using ranks (Spearman), the correlation was even stronger [0.53 (Ccr4), 0.47 (Dhh1), and 0.34 (Puf5)]. In contrast, correlations between the RIP-seq data from all three proteins and mRNA synthesis rates were weaker, and showed a modest negative-to-no correlation (Figure 4).

The synthesis rates measured by cDTA analysis were extrapolated from measured abundance and turnover rates (Sun *et al.* 2012). GRO-Chip analysis has been performed in yeast, which directly measures the transcription frequency across the genome (Molina-Navarro *et al.* 2008). To further verify that Ccr4 recruitment negatively correlated with mRNA synthesis rates, we examined the correlation between Ccr4 recruitment and mRNA synthesis rates measured by GRO-Chip and found a similar anticorrelation (Pearson = −0.25, Figure S8 in File S5). Therefore, using two different estimates of mRNA synthesis, Ccr4 recruitment to an mRNA negatively correlated with its synthesis rate.

Since Ccr4-Not and Dhh1 associate with elongating RNAPII (Kruk *et al.* 2011; Dutta *et al.* 2015), we considered that the recruitment that we detected may be linked to the process of transcription. To examine this possibility, we first looked for enrichment of these factors within intronic sequences. Yeast has 282 genes with introns (http://intron.ucsc.edu/yeast4.1/), and less than nine of these mRNAs showed enrichment of Ccr4, Dhh1, or Puf5 over both the exon and intron (Table S4 in File S5). Additionally, neither Ccr4 (*R* = −0.03) nor Dhh1 (*R* = −0.10) recruitment to mRNAs correlated with a published genome-wide ChIP-seq experiment that mapped the association of Ccr4-Not subunits with genes (Venters *et al.* 2011). Hence, few RNA–protein interactions detected here are likely to occur cotranscriptionally, and they are more likely to occur on cytoplasmic RNAs. Taken together, these results suggest that Ccr4-Not recruitment to mRNAs reflects its function in decay as opposed to synthesis.

A recent report suggested that the synthesis of ribosomal protein mRNAs may be regulated by Ccr4-Not in a Not5-dependent manner during stress (Gupta *et al.* 2016). However, we did not find a strong correlation with synthesis across the genome. The differences may be related to the subunits examined (NOTs *vs.* Ccr4 and Dhh1), the RNA enrichment strategy (native *vs.* cross-linked), or the positive correlation with transcription being restricted to a particular class of genes (see *Discussion*).

### mRNA decay factor recruitment anticorrelates with ribosome occupancy

A long-standing model posits that mRNAs are either partitioned into the translatable or nontranslatable pool, with the former category encompassing mRNAs that are associated with ribosomes and undergoing translation (Teixeira *et al.* 2005; Parker and Sheth 2007). We next explored the relationship between decay factor recruitment and the translation of mRNAs measured by Ribo-seq (Ingolia *et al.* 2009; Gerashchenko *et al.* 2012). Ccr4, Dhh1, and Puf5 recruitment displayed an anticorrelation with ribosome footprint data (R = −0.52, −0.42 and −0.33, respectively) (Figure 4). Thus, mRNAs bound to decay factors, especially Ccr4, are less likely to be translated at a high rate. This observation supports the long-standing model that deadenylation and translation are in competition (Parker and Sheth 2007), and is also consistent with the role of Dhh1 in translation repression (Presnyak and Coller 2013).

### Identification of Ccr4-associated mRNAs suggests that the two deadenylase complexes in yeast regulate different groups of mRNAs

Ccr4 and Pan2/3 are the main mRNA deadenylases in yeast, and two models have been proposed to explain how they cooperate in the decay of messages. The first model suggests that Pan2/3 initiates poly(A) shortening, which is then followed by complete deadenylation by Ccr4-Not (Brown and Sachs 1998; Tucker *et al.* 2001; Wahle and Winkler 2013). The second model, based on a study that found no correlation between the changes in decay rates of mRNAs in a *ccr4*∆ *vs.* a *pan2*∆ mutant, suggests that they each target different collections of mRNAs (Sun *et al.* 2013). Little is known about the interplay between these two complexes on the transcriptome; thus, we used the Ccr4 RIP-seq data to shed light on the contributions of Ccr4 and Pan2/3 to mRNA decay.

**Figure 5:**
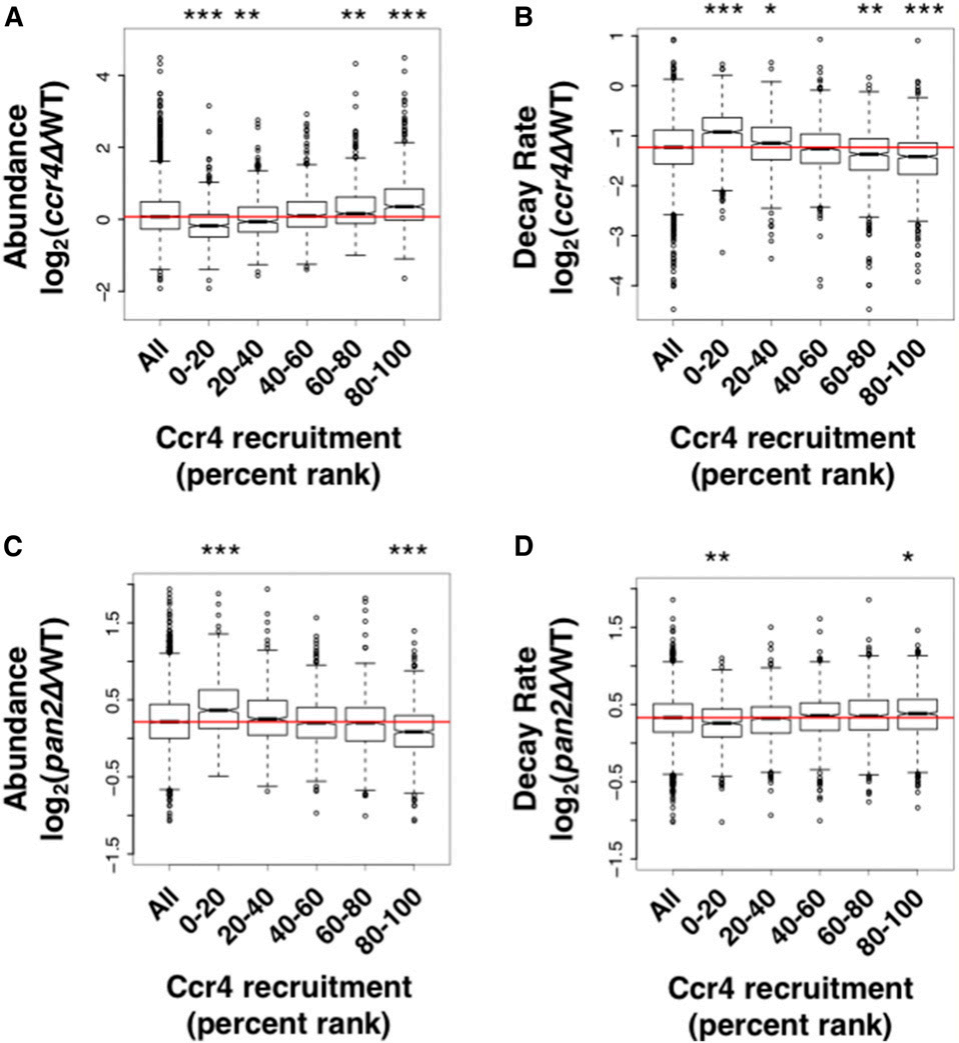
Identification of Ccr4 targets suggests a division of labor between the two cytoplasmic deadenylases. (A) Ccr4 RNA immunoprecipitation high-throughput sequencing (RIP-seq) enrichment values were equally divided into five groups based upon their percent rank (*i.e.*, 80–100 is the top 20th percentile). Each boxplot represents the log2 change in abundance in a *ccr4*∆ strain relative to a wild-type strain (Sun *et al.* 2013). Statistical significance was calculated using a Wilcoxon rank sum test with continuity correction. The red line indicates the median of all values. (B) The same as (A) but the boxplots represent the log2 change in decay rates in a *ccr4*∆ strain relative to wild-type. (C) The same as (A) but the boxplots represent the log2 change in expression in a *pan2*∆ strain relative to wild-type. (D) The same as (A) but the boxplots represent the log2 change in decay rates in a *pan2*∆ strain relative to wild-type. In order to clearly visualize the boxplots in (A), (C), and (D), two outlier data points were removed from the *y*-axis. * p-value < 0.001, ** p-value < 0.0001, and *** p-value < 2.2 × 10^−16^.

The Ccr4-enriched mRNAs were divided equally into five percentile ranks, and boxplots were created using the relative abundance values in a *ccr4*∆ mutant *vs.* a wild-type strain (Figure 5A). This plot illustrates that the most highly enriched mRNAs increased in abundance in a *ccr4*∆ deletion (top 20%, p-value < 2.2 × 10^−16^), and the lowly (essentially no recruitment) enriched mRNAs actually decreased in abundance (bottom 20%, p-value < 2.2 × 10^−16^). Although almost all mRNAs had decreased decay rates in the *ccr4*∆ mutant, mRNAs that were highly enriched by Ccr4 had the largest decrease in their decay rate (p-value < 2.2 × 10^−16^, Figure 5B), and those that were lowly recruited had the smallest decrease in their decay rate (Figure 5B). Similar correlations were also observed between Dhh1 enrichment and changes in abundance and decay in a *dhh1*∆ mutant (Figure S9, A and B in File S5).

mRNAs that were stabilized in a *pan2*∆ mutant were either not strongly affected or were turned over more rapidly in a *ccr4*∆ strain (Sun *et al.* 2013), suggesting that Ccr4 and Pan2/3 may regulate different mRNAs. Consistent with this, we found that Ccr4-enriched transcripts displayed reduced abundance (p-value < 2.2 × 10^−16^, Figure 5C) and increased decay rates (p-value < 0.001, Figure 5D) in the *pan2*∆ mutant. Also, mRNAs that were lowly enriched increased in abundance (p-value < 2.2 × 10^−16^, Figure 5C) and displayed reduced decay rates (p-value < 0.001, Figure 5D) in the *pan2*∆ mutant. In other words, the abundance and stability of Ccr4-bound mRNAs are most affected by the deletion of *CCR4*, while the lowly enriched targets are more strongly affected by the deletion of *PAN2*. The decrease in abundance of Ccr4 targets in the *pan2*∆ mutant is interesting. The cause of this is not known but it suggests that, in the absence of Pan2, either Ccr4 compensates for its loss by increasing its activity or another redundant pathway, such as the exosome, becomes more prevalent.

### Ccr4 and Dhh1 bind mRNAs that respond to the metabolic state of the cell

**Figure 6:**
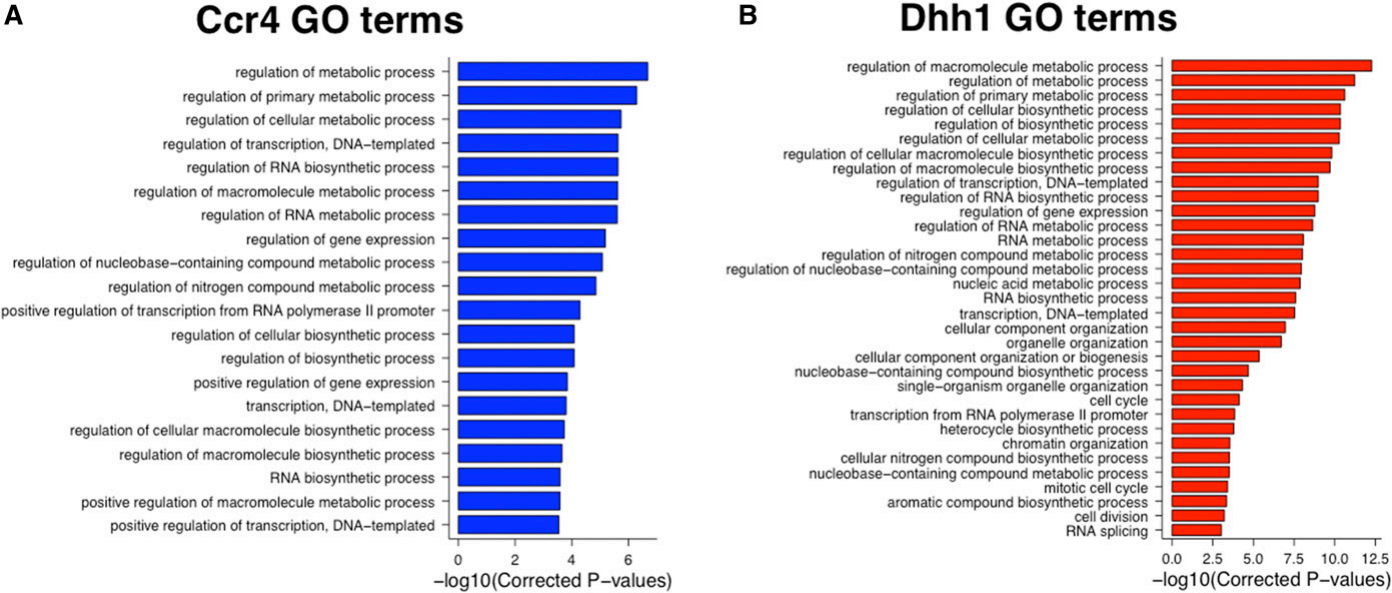
Ccr4 and Dhh1 are recruited to mRNAs involved in metabolic processes (A). Gene ontology (GO) terms analysis of the top 20th percentile of Ccr4-enriched RNAs (*N* = 846 genes). (B) The same as (A), except that the top 20th percentile of Dhh1-enriched RNAs (*N* = 773 genes) was analyzed.

To identify the pathways regulated by Ccr4, Dhh1, and Puf5, lists of the most highly enriched mRNAs (top 20%) were submitted to GO terms analysis. The Puf5-bound mRNAs had terms associated with transcription and RNA biogenesis overrepresented, among others (Figure S10 in File S5). This is consistent with a previous native RIP-Chip study, which also revealed that Puf5 targets transcriptional and chromatin regulators (Gerber *et al.* 2004). The mRNAs that showed the highest level of recruitment to Ccr4 and Dhh1 code for proteins involved in many of the same processes, such as RNA metabolism, nitrogen compound metabolism, transcription, and several biosynthetic processes (Figure 6, A and B). The identification of many of the same GO terms in the Ccr4 and Dhh1 data sets is consistent with the high degree of overlap between the mRNA targets of these two proteins (Figure 2, A and B). Dhh1 was also recruited to 232 mRNAs important for organelle organization processes (Figure 6B). This collection of mRNAs was further analyzed using GO Slim Mapper to narrow down the organelle(s), which revealed that 26% of the messages were specifically connected to mitochondrial organization (File S4).

**Figure 7:**
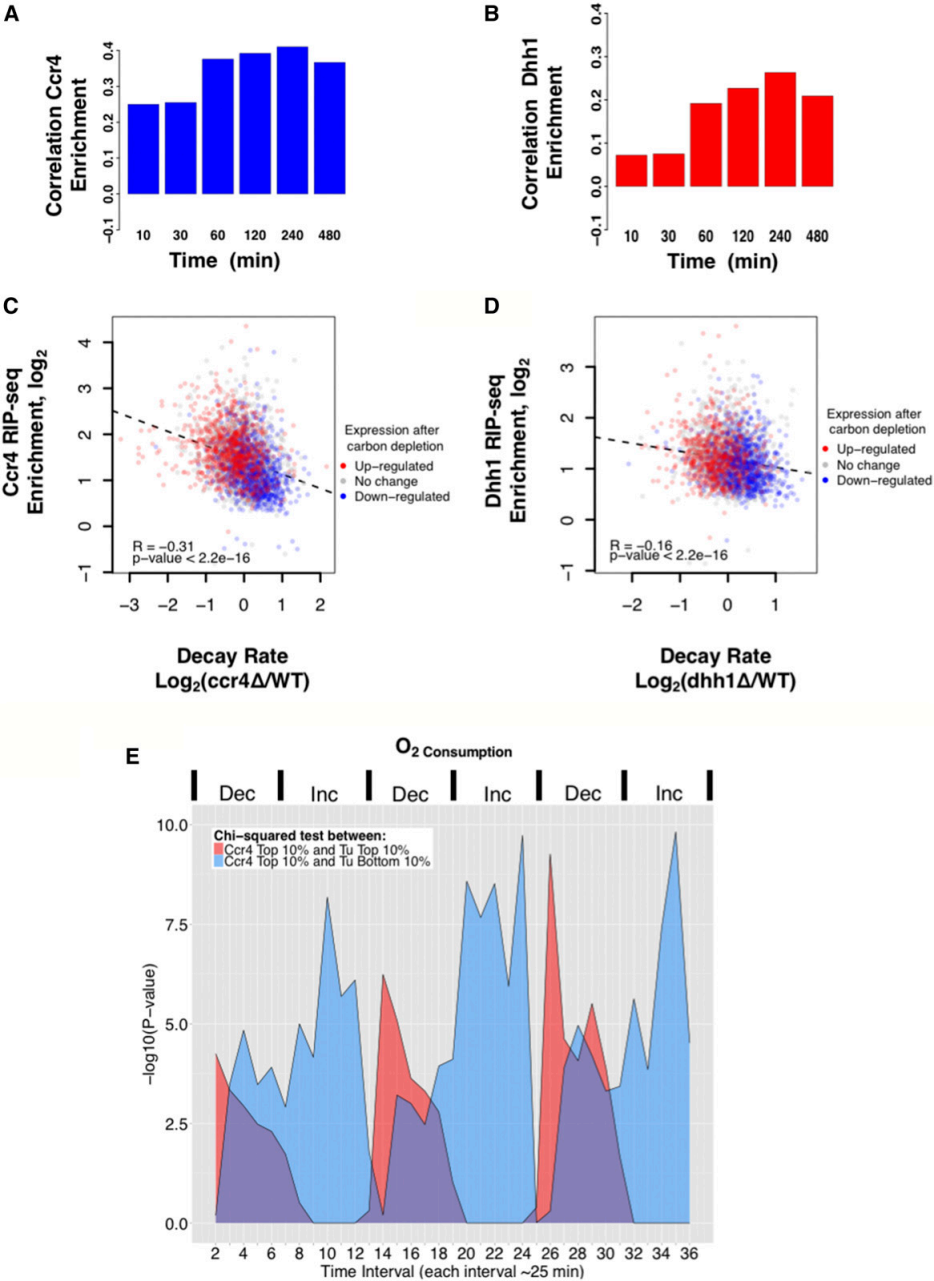
Ccr4 and Dhh1 are recruited to mRNAs that respond to nutrient and metabolic signals. (A) Pearson correlation values were calculated between Ccr4 RNA immunoprecipitation high-throughput sequencing (RIP-seq) enrichment and expression values from the carbon depletion experiment in Bradley *et al.* (2009). Each column represents a different time point after carbon depletion relative to the unstressed condition. (B) The same as in (A), except that Dhh1 RIP-seq enrichment values were used. (C and D) Scatter plots comparing RIP-seq enrichment to the change in decay rates in a deletion strain relative to wild-type. The plots for Ccr4 (C) and Dhh1 (D) are shown. The color of each data point represents how the expression changed at the 240 min time point after carbon depletion from Bradley *et al.* (2009). Genes that were upregulated (log2 expression > 1), downregulated (log2 expression < −1), or unchanged were colored red, blue, and gray, respectively. The dashed black line trend line represents the least squares line of regression. (E) A χ^2^ test was performed between the highly enriched Ccr4 targets (top 10th percentile) against the upregulated genes [top 10th percentile of genes (red area)] and downregulated genes [bottom 10th percentile (blue area)] from Tu *et al.* (2005). The periods when oxygen consumption was increasing (Inc) or decreasing (Dec) is displayed above the graph.

Since mRNAs involved in multiple metabolic processes were identified in the RIP-seq data sets, we examined the correlation between our recruitment data and gene expression changes during metabolic stress (Bradley *et al.* 2009). We found that the most highly enriched mRNAs (top 10th percentile) bound to Ccr4 and Dhh1 overlapped significantly with genes that are induced during nitrogen and carbon starvation (Figure S11, A and B in File S5; p-values from < 0.001 to < 1 × 10^−14^). In order to establish if this relationship reached beyond the top 10th percentile of targets, we calculated the correlation between RIP-seq data and the changes in gene expression over time during carbon and nitrogen starvation (Figure 7 and Figures S11 and S12 in File S5). Ccr4 showed positive correlations between its enrichment values and gene expression levels during carbon starvation (Figure 7A). Furthermore, the Pearson correlation values increased as cells progressed deeper into the starvation program, which most likely results from the accumulation of more genes with expression changes over time (Bradley *et al.* 2009). Although Dhh1 enrichment did not correlate with gene expression changes during nutritional stress as strongly as Ccr4 did, the same trends between recruitment and gene expression changes were still observed (Figure 7B and Figures S11 and S12 in File S5).

We found that Ccr4 and Dhh1 recruitment correlated with changes in gene expression during carbon source deprivation stress. We next attempted to determine how many of these differentially expressed mRNAs are dependent upon Ccr4 and Dhh1, and to what extent the correlations are driven by changes in the decay of these messages. We constructed scatter plots that displayed mRNA enrichment (*y*-axis) *vs.* changes in decay rates (*x*-axis) in the mutants. Each point was then colored red, blue, or gray to indicate if the mRNA expression increased, decreased, or remained unchanged in stressed cells, respectively. The plot shows that the upregulated mRNAs were predominantly those that showed the highest Ccr4 recruitment and the largest decrease in decay rate in the *ccr4*∆ mutant (Figure 7C, *R* = −0.31, p-value < 2.2e−16). Conversely, the genes that were downregulated more often had transcripts that displayed a modest increase in decay rate and were less enriched with Ccr4 compared to upregulated genes. The same trend was observed for Dhh1 (Figure 7D), but to a lesser extent (*R* = −0.16). Finally, repeating the same analysis in cells undergoing nitrogen depletion stress also revealed the same pattern (Figure S12 in File S5). In summary, the genes that are differentially expressed during metabolic stress are regulated during unstressed conditions by Ccr4 and Dhh1, which likely involves changes in mRNA stability.

Methods of carbon and nitrogen depletion differ between studies, as do other variables. For example, the study by Bradley *et al.* (2009), which was used as the data set for our analysis, depleted nutrients by transferring cells on membranes between different media conditions (in this case, nitrogen base plates replete of depleted of nutrients). To demonstrate that our conclusions are robust and not unique to any depletion technique or condition, we repeated our analysis using data sets from two other studies measuring gene expression changes during nutrient limitations (Figure S11C in File S5). One study that measured the response to nitrogen depletion shifted cells from liquid culture in nitrogen-containing to nitrogen-depleted medium (Gasch *et al.* 2000). Another measured changes in gene expression by shifting cells in rich (YPD) liquid media to medium lacking dextrose (Arribere *et al.* 2011). The latter condition is most like those used to grow cells in this work. Repeating the analysis using these other data sets lead to the same conclusion: the RIP-seq data correlated with the changes in gene expression during carbon and nitrogen starvation (Figure S11C in File S5). Thus, we found similar correlations between the RIP-seq targets and changes in gene expression from three different studies, suggesting that the correlations we identified are robust and not unique to any one depletion protocol, data set, or strain used to generate the expression data.

The identification of many mRNA targets associated with metabolic processes prompted us to investigate the relationship between mRNA decay machinery recruitment and changes in gene expression during the YMC. The YMC is a process in which budding yeast cells oscillate between the storage (reductive, nonrespiratory phase) and consumption (oxidative, respiration) of metabolites, which produces waves of gene expression that correlate with bursts of O_2_ utilization (Tu *et al.* 2005; Cai and Tu 2012). The overlap between the most highly enriched Ccr4 targets (top 10%) and mRNAs that were highly or lowly expressed during the YMC was calculated, a χ^2^ test was performed, and the p-values of the overlap were plotted *vs.* time (Figure 7E). The top Ccr4 targets significantly overlapped (p-value < 10^−4^) with the most highly expressed genes when cells decreased their O_2_ consumption during the nonrespiratory phase (Figure 7E, see time points 2, 14–15, and 26–28). Strikingly, as cells progressed out of a nonrespiratory phase and into the respiratory phase (increasing O_2_ consumption), the top Ccr4 targets significantly overlapped (p-value < 10^−4^) with the strongly repressed genes (bottom 10%) (Figure 7E, see time points 8–12, 19–24, 32, and 34–36). These patterns suggest that Ccr4 may target specific transcripts in response to metabolic signals that arise from a change in the redox or metabolic state of the cell. Comparing the overlap between Dhh1 targets and fluctuations in gene expression produced similar trends, although the level of overlap, based on p-values, was less extensive than those observed for Ccr4-associated transcripts (Figure S12A in File S5). In contrast, there was no significant overlap between Puf5 targets and the genes fluctuating during the YMC (Figure S12B in File S5). Interestingly, the Puf3 motif was overrepresented both among the top Ccr4 targets identified in our study (Figure 3B) and among the genes that showed peak expression during the transition from increased to decreased oxygen consumption (Tu *et al.* 2005). This suggests that Puf3, a protein shown to recruit Ccr4-Not to a single mRNA encoding a protein involved in regulating mitochondrial respiration, COX17, (Lee *et al.* 2010), regulates the association of Ccr4-Not with many transcripts during the YMC.

## Discussion

The integration of mRNA expression data (*e.g.*, microarrays) with transcription factor recruitment throughout the genome (ChIP-Chip or ChIP-seq) has been an important advance in our understanding the mechanisms of transcription regulation. For instance, correlating the binding of RNA polymerase II to genes with changes in mRNA levels has identified direct effects and provided deeper mechanistic insights into the process of gene transcription. In contrast to transcriptional regulators, which have been studied extensively, much less is known about the relationship between the binding of mRNA regulatory factors and the fate of mRNAs on a global scale. Our study identified the targets of three characterized mRNA decay factors, Ccr4, Dhh1, and Puf5, and these data were used to address their roles in the control of gene expression.

### Identifying mRNA targets of Ccr4 provides insights into its role in transcription and decay

The Ccr4-Not complex regulates mRNA levels by directly regulating synthesis in the nucleus and mRNA decay in the cytoplasm (Miller and Reese 2012). Expression profiling in Ccr4-Not mutants has been performed (Cui *et al.* 2008; Azzouz *et al.* 2009; Sun *et al.* 2013), but disentangling direct *vs.* indirect effects and attributing a change in mRNA levels to altered synthesis or decay is not trivial without a global map of Ccr4–mRNA interactions. We utilized existing genome-wide mRNA synthesis and decay rate data to illustrate that Ccr4 recruitment, and that of its binding partners Dhh1 and Puf5, correlated better with decay rather than synthesis. In fact, the highly enriched mRNAs displayed a lower synthesis rate overall. Considering that Ccr4-Not directly promotes elongation *in vitro* (Kruk *et al.* 2011; Babbarwal *et al.* 2014; Dutta *et al.* 2015), cross-links to active genes (Kruk *et al.* 2011; Venters *et al.* 2011; Gupta *et al.* 2016), and was first identified as a transcription factor (Collart 2003; Miller and Reese 2012), its anticorrelation to synthesis rates was surprising on the surface. However, an explanation for this observation is that Ccr4-Not is utilized more at lowly synthesized genes that are under greater elongation control.

Ccr4 is the main cytoplasmic deadenylase in eukaryotes and plays a global role in decay. Therefore, it might be expected that it is bound to nearly every mRNA. We found this not to be the case. There are a number of factors, some technical and others biological, which can explain this. Technically, the stringent 1% FDR filtering and imposition of a twofold cut off would exclude some mRNAs from the data set. A biological explanation is redundancy with the other deadenylase, Pan2. Comparison of the level of Ccr4 recruitment to the changes in gene expression in *ccr4*∆ and *pan2*∆ mutants showed that mRNAs that are not/lowly bound to Ccr4 are more strongly affected by the deletion of *PAN2* (Figure 5). This is highly suggestive of redundancy between these two deadenylases. There may be a contribution from deadenylation-independent decay pathways as well, such as exosome-mediated decay (Schmid and Jensen 2008).

### Analysis of decay factor targets does not support a widespread imprinting mechanism as a major mode of action to regulate mRNA levels

As stated above, decay factors such as Ccr4-Not, Dhh1, Xrn1, Lsm1, and Dcp2 cross-link to the promoters and open reading frames of highly transcribed genes (Lin *et al.* 2008; Kruk *et al.* 2011; Haimovich *et al.* 2013b; Faraji *et al.* 2016). These findings, along with others, have led to speculation that mRNAs could be imprinted with decay factors during transcription in the nucleus, where they mark mRNAs for post-transcriptional regulation and decay in the cytoplasm (Haimovich *et al.* 2013a; Reese 2013). Ccr4-Not is a good candidate to carry out this function, since it participates in both the synthesis and destruction of mRNAs (Miller and Reese 2012; Reese 2013; Collart 2016; Gupta *et al.* 2016). If the imprinting of Ccr4 onto mRNAs during transcription was an obligate step in the targeting of mRNAs for regulation in the cytoplasm, one might expect that Ccr4 would be loaded onto the vast majority of newly synthesized RNAs proportional to their synthesis; thus, Ccr4 recruitment would correlate positively with transcription rates. As discussed above, this was not the case. These results do not rule out the possibility that Ccr4-Not is loaded onto mRNAs cotranscriptionally and escorts them out into the cytoplasm, but once in the cytoplasm its binding to mRNAs may be subject to dynamic regulation driven by normal cellular cues and/or redistribution to specific mRNAs by sequence-specific mRNA-binding proteins.

A recent paper described a genome-wide map of Not1 targets (Gupta *et al.* 2016). The authors of that paper concluded that mRNAs are imprinted with Not1 in the nucleus, which affects the ultimate translation of the mRNA. This appears to apply to a subset of genes, such as those encoding ribosomal proteins. We found some similarities between the Ccr4 targets described here and Not1 targets identified in the other study. For example, both Ccr4 and Not1 associated with mRNAs with a Puf3 motif and were bound by other RBPs known to genetically or physically interact with Ccr4-Not (Gupta *et al.* 2016). Nonetheless, the correlation between the Ccr4 and Not1 RIP-seq data was weak (Spearman’s *R* = 0.11, Figure S13A in File S5), and our Ccr4 recruitment data more strongly correlated with global mRNA decay rates (*R* = 0.53) than the Not1 data did (*R* = 0.06) (see Figure 4 and Figure S13B in File S5). Additionally, we found a smaller number of overlapping (562 mRNAs) targets than anticipated, considering that the two proteins reside in the same complex and results indicate that Ccr4 must bind to Not1 to carryout mRNA decay (Basquin *et al.* 2012). Having said that, technical differences between the studies may explain this. Most notably, we used rapid formaldehyde cross-linking and detergents in the isolation step, which allows for more stringent determination of targets, and would prevent the redistribution of RBPs and inhibit degradation of mRNAs during isolation (Riley and Steitz 2013). As we have found to be the case for Dhh1, studies not using a cross-linking step (Hogan *et al.* 2008; Cary *et al.* 2015) identified far fewer targets than those that did [ours and that of Mitchell *et al.* (2013)]. Another significant difference is the proteins examined. We examined Ccr4 and Dhh1, proteins that form the nuclease module of the complex. It is possible that Not1 has a different behavior than the nuclease submodule subunits analyzed here. Gupta *et al.* (2016) demonstrated by immunofluorescence that Not1 staining is largely nuclear, while multiple studies have shown that Ccr4 and Dhh1 are predominantly cytoplasmic (Tucker *et al.* 2001; Fischer and Weis 2002; Mitchell *et al.* 2013). Furthermore, Ccr4 (and Dhh1) bind the N-terminus of Not1 and display different genetic interactions than those that bind to the C-terminus of Not1, such as Not5 (Collart 2003). Since there is the potential for heterogeneity among Ccr4-Not complexes in yeast and metazoans [for review see Miller and Reese (2012)], it is possible that separate pools of Not1 (with Not5 and others) are dedicated to global decay and imprinting.

### mRNAs highly enriched with decay factors are less likely to have high ribosome occupancy

A long-standing model for the fate of mRNAs is that they are partitioned into either the translatable or nontranslatable pool, with the latter category encompassing mRNAs that are stored in mRNPs or undergoing degradation (Teixeira *et al.* 2005; Parker and Sheth 2007). However, recently, the lines have become blurred. The processes of translation and decay is more intertwined than was initially thought, and some mRNA decay factors have also been implicated in regulating translation (Coller and Parker 2005; Chritton and Wickens 2010; Pelechano *et al.* 2015; Collart 2016; Gupta *et al.* 2016). In addition, decay factors and 5′ to 3′ decay intermediates have been detected among polyribosomes (Hu *et al.* 2009; Sweet *et al.* 2012; Pelechano *et al.* 2015; Preissler *et al.* 2015), suggesting that RNAs undergoing translation can be subjected to decay.

We found that decay factor recruitment negatively correlated with ribosome density. If deadenylation mostly occurred on polyribosomes, it would be expected that the recruitment of the major initiator of decay, Ccr4, would show a positive correlation with ribosome density. This was not the case. Furthermore, the mRNA targets of the decay factors analyzed here correlated with those targets of multiple proteins involved in decay and translational repression, and not those that promote translation. The relationship between decay factor recruitment and ribosome occupancy described here, and the correlations between the deadenylase and other repressors of translation, supports a model that deadenylation and translation of transcripts are in competition. It is important to note that our results do not eliminate the existence of cotranslational decay pathways. Additional studies are required to fully understand the relationship between translation and decay.

### Targeting of Ccr4 and Dhh1 to nutrient-regulated transcripts suggests that mRNA decay and/or translational repression are important for cells to respond to nutrient availability and environmental conditions

*CCR4* was initially identified as a regulator of *ADH2* and other nonfermentative growth genes (Denis 1984). This discovery provided the first evidence that Ccr4 plays an important role in the cell’s ability to adjust to carbon sources. Genetic studies have revealed that Ccr4 and Dhh1 are required for cells to survive nutrient starvation (Westmoreland *et al.* 2003, 2004; Bergkessel and Reese 2004), and the Dhh1 homolog from *Schizosaccharomyces pombe*, ste13+, regulates N_2_ starvation-induced entry into meiosis (Maekawa *et al.* 1994). Furthermore, Ccr4-Not and Dhh1 have been tied to the TOR pathway (Talarek *et al.* 2010; Laribee *et al.* 2015). Thus, there is previous genetic evidence that Ccr4-Not is an important regulator of the nutrient response. Our work has added to these observations and provided additional insights into how Ccr4 and Dhh1 regulate gene expression in response to nutrient levels. Ccr4 and Dhh1 associate with mRNAs that are normally repressed in rich media and induced by nutrient starvation. Significantly, these mRNAs are under the tightest control of these factors at the level of decay (*i.e.*, highly enriched and stabilized by deletion of the gene) (Figure 7, C and D). This suggests a logical model whereby Ccr4 and Dhh1 are recruited to these mRNAs when nutrients are rich to repress their expression by mediating the decay of these messages. However, as stated in other parts of this manuscript, Ccr4-Not regulates gene expression at the level of transcription, binds transcription factors, and is recruited to active genes. Therefore, its functions in the nutritional response are unlikely to be restricted to mRNA decay. However, analysis presented here, and that of others, presents a solid case that Ccr4 and Dhh1 are important for the cell to control nutrient-regulated genes, and that this involves regulation of the decay of the messages.

Typically, experiments are carried out under exaggerated conditions (*e.g.*, nutrient-rich *vs.* complete starvation), comparing two “static” conditions to amplify effects. Although contrasting nutrient-rich and -poor conditions is useful, understanding how yeast behave during the YMC has been suggested as a better way to capture the reprograming of metabolic pathways (Cai and Tu 2012). Thus, the more interesting result of our mapping study is the uncovering of the previously unknown relationship between Ccr4 and Dhh1 recruitment and the fluctuations of mRNAs during the YMC. We found that Ccr4 and Dhh1 are highly recruited to mRNAs that are repressed as cells move from the nonrespiratory phase into the respiratory phase (increasing O_2_ consumption). As mentioned in other sections of this manuscript, Ccr4-Not is targeted to specific mRNAs via RBPs. The Puf3 motif is overrepresented in the Ccr4-bound mRNAs (Figure 3) and also in those that fluctuate during the yeast YMC (Tu *et al.* 2005; Lee and Tu 2015). Puf3 immunoprecipitates with Ccr4, suggesting that a physical interaction between Puf3 and Ccr4-Not is important for deadenylase recruitment (Lee *et al.* 2010). Although Ccr4 and Dhh1 are bound to messages undergoing nutrient control, levels of Ccr4, Dhh1, or Puf5 proteins do not change under nutrient starvation conditions or when cells are grown in nonfermentable carbon sources (Figure S14A in File S5). Whether nutrient signals affect the recruitment of Ccr4-Not to mRNAs or regulate its activity remains to be tested. Not1, Not4, and Caf1 are phosphoproteins, and Not4 becomes phosphorylated in stressed cells (Lau *et al.* 2010) and Caf1 undergoes phosphorylation in carbon-starved cells (Moriya *et al.* 2001). We noticed that the mobility of Not4, a subunit of the complex, changed when cells were starved for carbon or grown in nonfermentable carbon sources (Figure S14B in File S5). Puf3p phosphorylation during the YMC and glucose starvation correlated with increased accumulation and decreased turnover of Puf3-targeted mRNAs (Tu *et al.* 2005; Miller *et al.* 2014; Lee and Tu 2015). Taken together, these observations raise the intriguing possibility that Ccr4-Not, and/or the proteins that recruit it to messages, are regulated by the nutritional status of the cell. Testing this hypothesis is worthy of comprehensive future studies, which are well beyond the scope of the work reported here.

The association of decay factors with specific messages in response to metabolic fluxes may be required to rapidly “clear” the cell of these mRNAs, allowing the cell to transition between phases of fermentation and respiration. In addition, recruitment of decay factors could sharpen peaks of gene expression to impart tighter temporal control over the YMC, similar to how changes in the decay and synthesis rates of cell cycle-regulated messages are important during the mitotic cell cycle (Eser *et al.* 2014). Previous genetic studies indicate that Ccr4 and Dhh1 are required for cells to survive stresses that affect the cell cycle (Westmoreland *et al.* 2003, 2004; Bergkessel and Reese 2004). Thus, the regulated targeting of decay factors to transcripts is likely to be important for cells to respond to and recover from stressful conditions.

## Supplementary Material

Supplemental material is available online at www.g3journal.org/lookup/suppl/doi:10.1534/g3.117.300415/-/DC1.
