# Supplementary material for: Genome-Wide Mapping of Decay Factor–mRNA Interactions in Yeast Identifies Nutrient-Responsive Transcripts as Targets of the Deadenylase Ccr4
Source: G3 (Bethesda). 2017 Nov 20;8(1):315–30. doi: 10.1534/g3.117.300415 (PMC5765359; doi:10.1534/g3.117.300415)
Supplement: Supplementary file 5 [file 315FileS5.pdf]

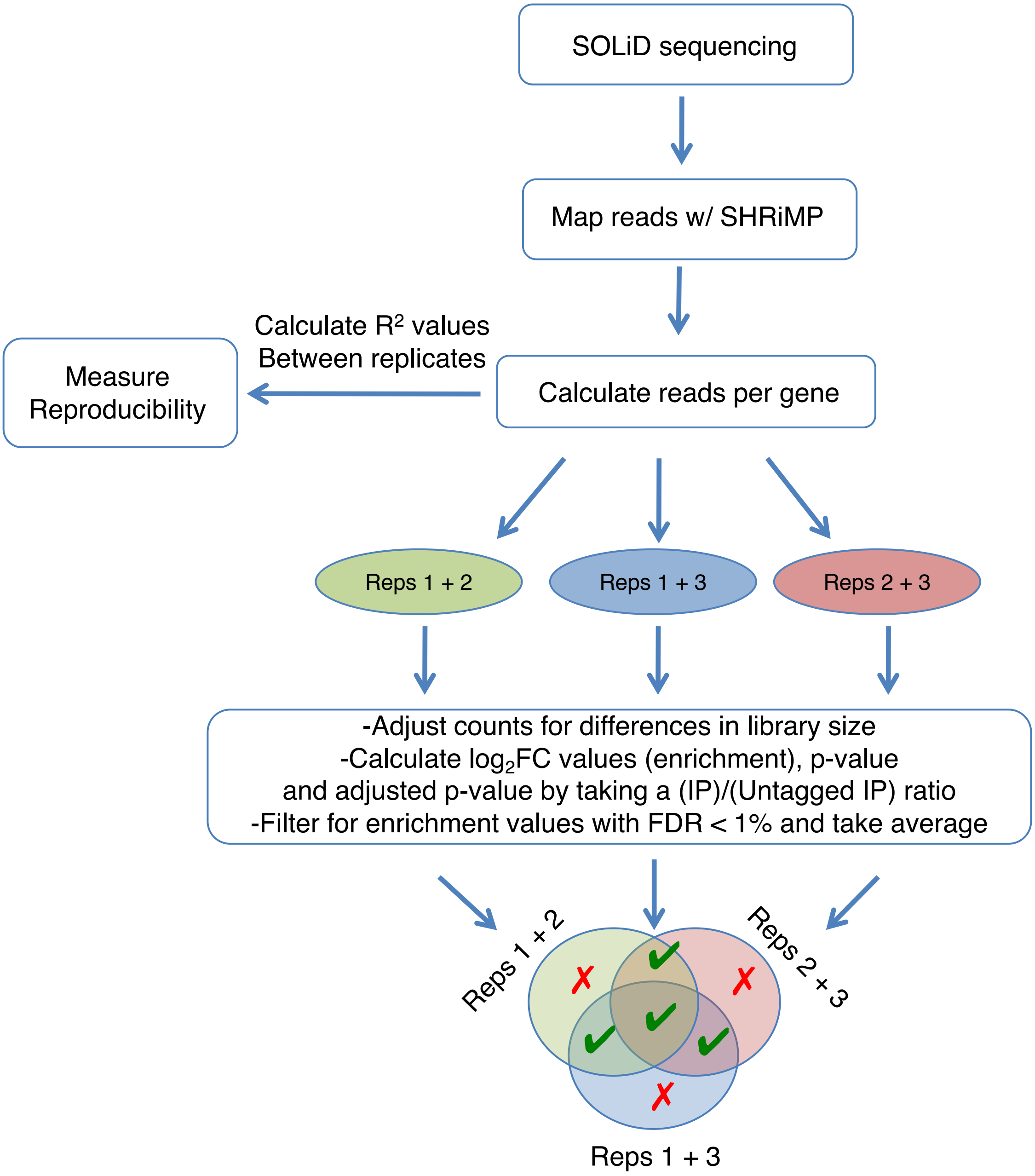

Figure S1: Bioinformatics workflow for calculating enrichment values. The reads from each replicate were downloaded separately by barcode. Reads were trimmed 15bp from the 3' end as a quality control measure, and then mapped to the *S. cerevisiae* genome (sg7) using SHRiMP version 2.2.2. The number of uniquely mapped reads within annotated transcripts (David et al. 2006) was calculated for each gene. These values were used for measuring reproducibility (Table S1). Enrichment of an RNA with a given protein (uniquely mapped reads per gene of the IP divided by the mock IP reads) was calculated in edgeR (see methods for details). The reads per gene were adjusted for differences in library size between samples. P-values were adjusted for multiple testing using the Benjamini & Hochberg method (FDR). In order to increase the level of confidence in the identification of the RNAs bound to each protein, genes that had enrichment values with FDR < 1% using at least two of the three combinations (i.e. reps 1+2 and reps 2+3) were identified and used.

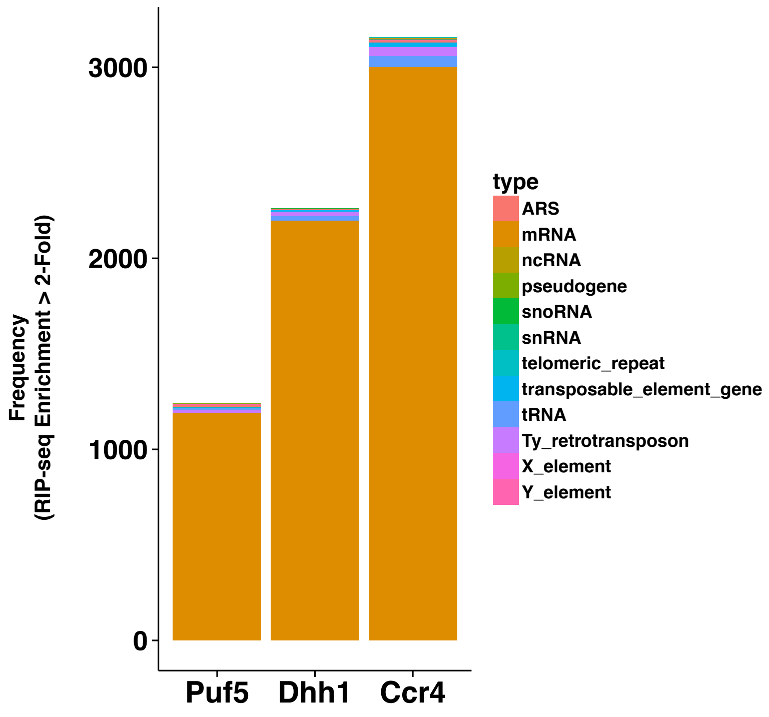

Figure S2
Number of different types of RNAs enriched at least two-fold (>1 $\log_2$).

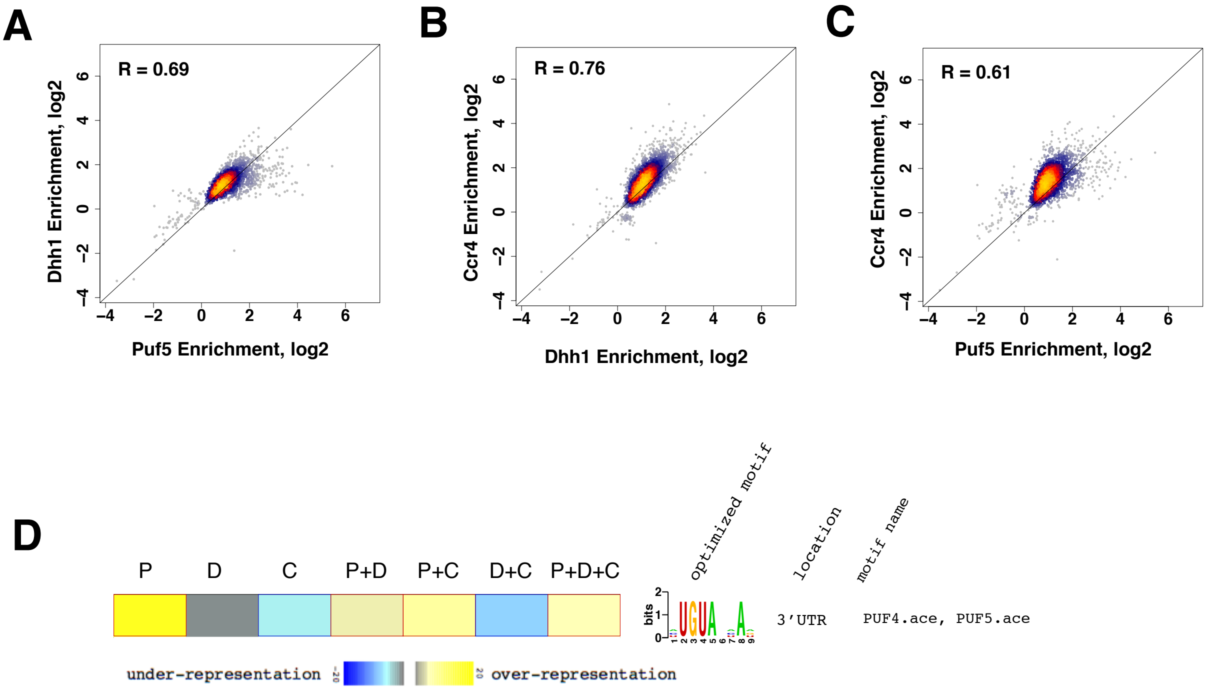

Figure S3: Scatter-plot and motif analysis of RIP-seq enrichment values
(A-C) Pairwise comparison between enrichment values meeting the FDR 1% cutoff. The Pearson correlation value is displayed in the box. The black diagonal line represents a slope equal to 1. The more yellow the data point, the higher the density of points around it, where as the more gray the less dense the area is with data points. (D) The RNAs from the venn diagram in Figure 2B were submitted to FIRE (Elemento et al. 2007) in terms of P (only Puf5 was enriched), D (only Dhh1 was enriched), C (only Ccr4 was enriched), P+C (only Puf5 and Ccr4 were enriched), P+D (only Puf5 and Dhh1 were enriched), D+C (only Dhh1 and Ccr4 were enriched) and P+D+C (RNAs where all three proteins were enriched). The color bar below represents the p-value for each bin, where yellow refers to the motif being over-represented and blue being under-represented. FIRE produced the optimized motif, and identified the location and name.

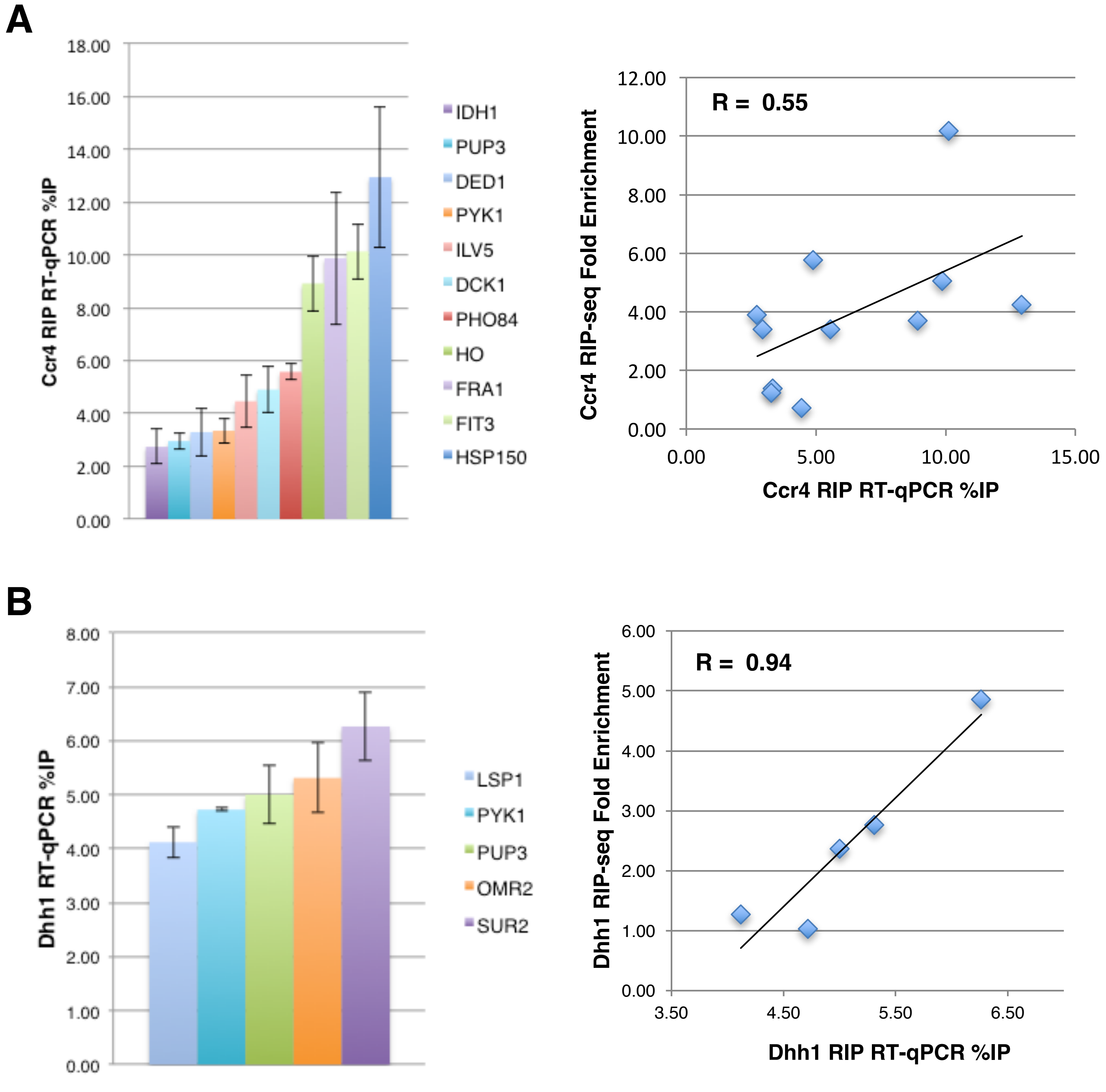

Figure S4: Validation of RIP-seq with RIP RT-qPCR

Validation of RIP-seq using RIP RT-qPCR analysis of several targets of Ccr4 (A) and Dhh1 (B). The RT-qPCR data represents the percentage IP (IP signals/input signals) and is shown as the average and standard deviations of three biological replicates. The scatterplots to the right compare RT-qPCR results with RIP-seq. Pearson correlations are shown in the upper left hand corner of each plot.

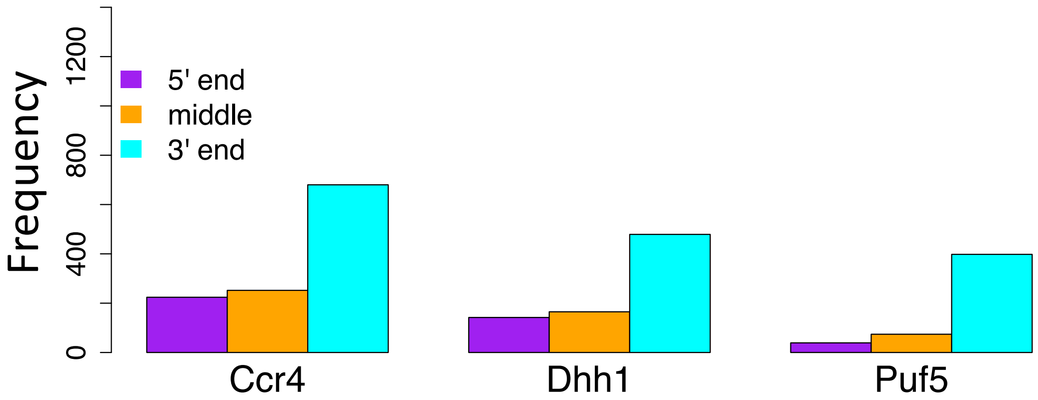

Figure S5. Distribution of factor recruitment based on RNA lengths. mRNAs (>400 nt) were divided into thirds (5’ end, middle and 3’ end) and enrichment in each segment was calculated as described in Figure S1 and in the methods section. The frequency of an mRNA >4-fold enrichment is displayed on the y-axis. mRNAs where enrichment was observed at more than one region (i.e. the middle and 3’ end) are not included in the graph.

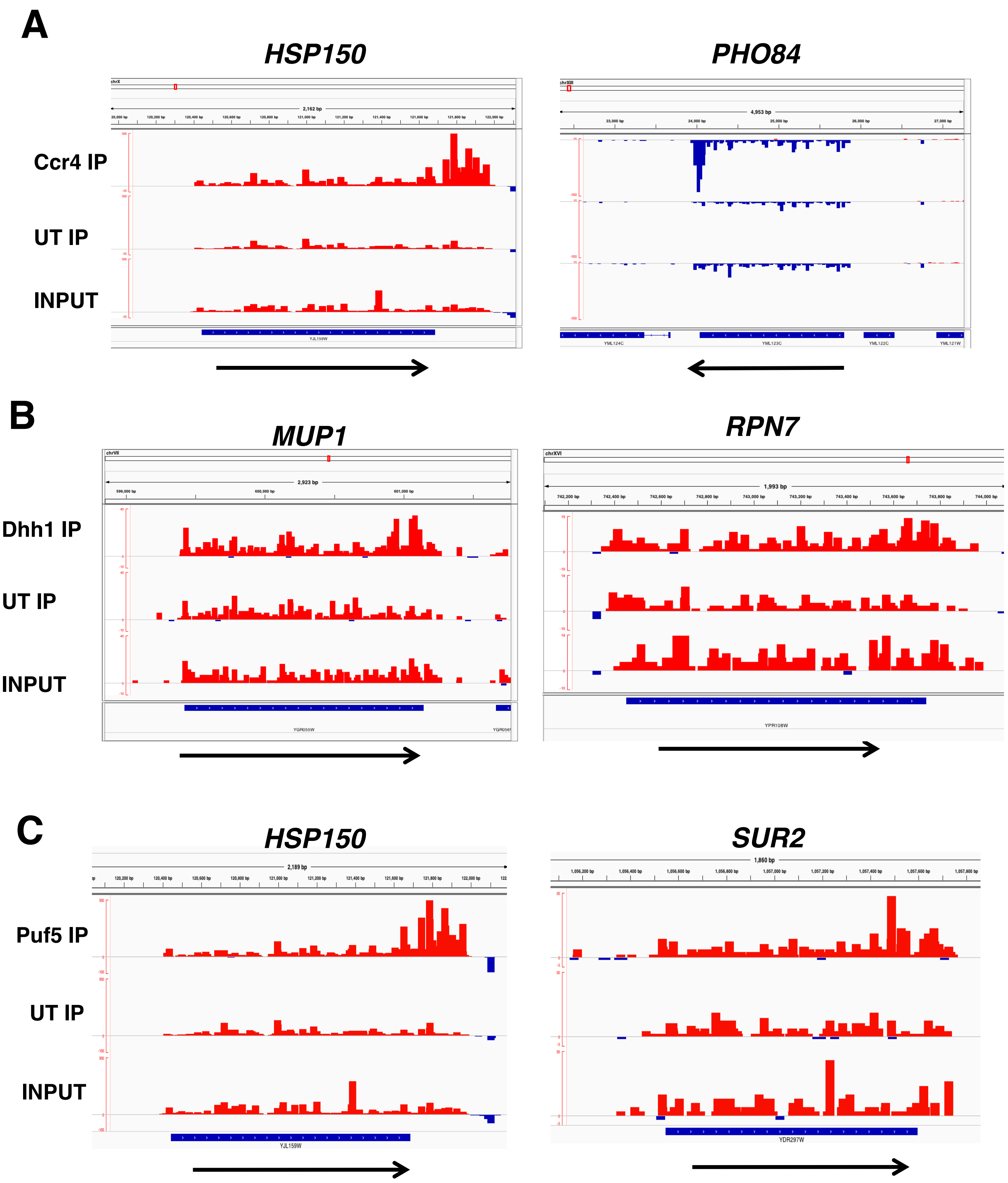

Figure S6: Screen shots of mRNAs. (A) Ccr4, (B) Dhh1 and (C) Puf5. Reads were combined from all replicates for each screen shot then adjusted to have equivalent library sizes and visualized in the program IGV (Integrated Genomics Viewer). Reads that mapped to the positive or negative strand are depicted in red and blue bars, respectively. The black arrow below the screen shot illustrates the 5’ to 3’ direction of the gene of interest.

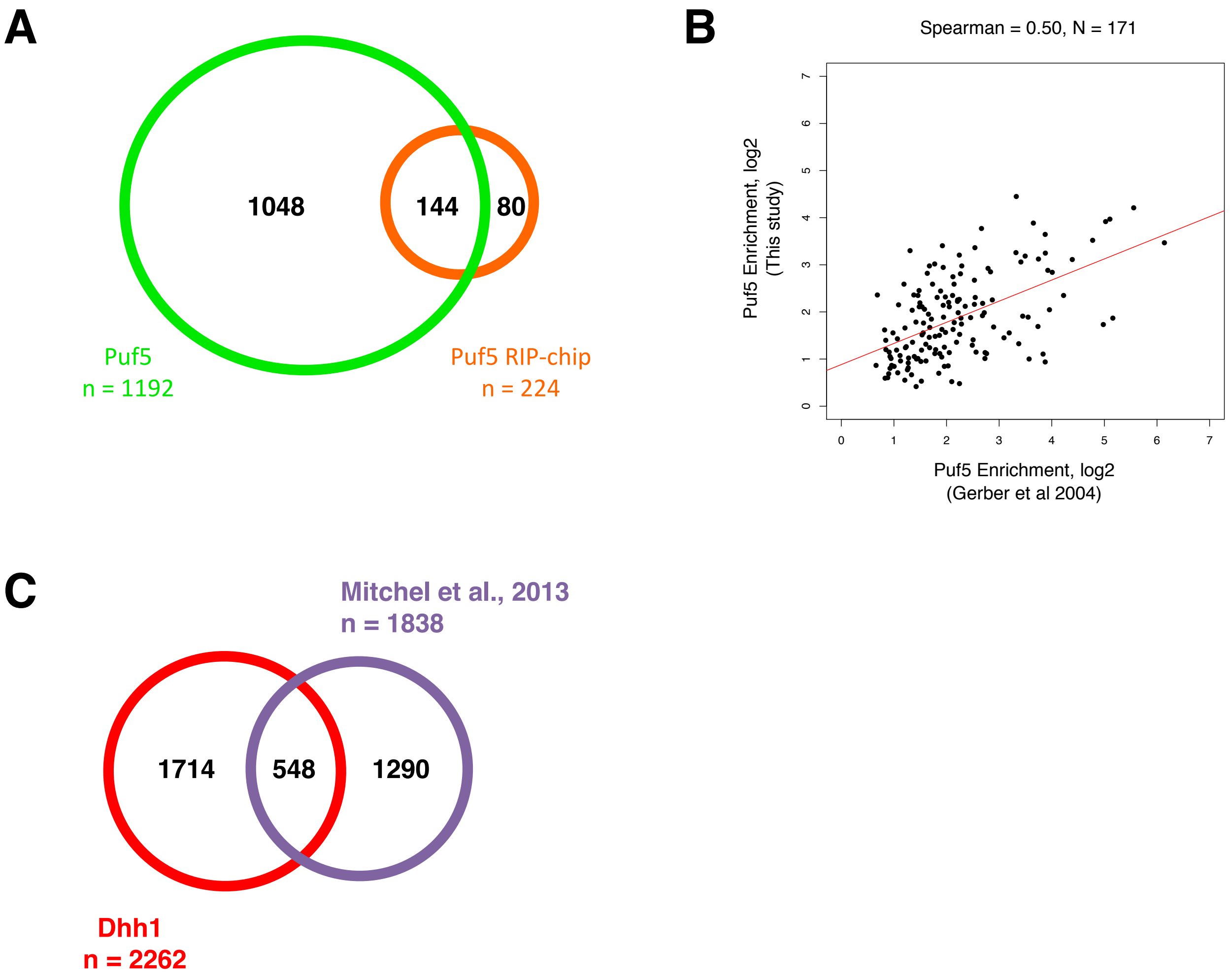

Figure S7: Overlap between RIP-seq and previous studies
(A). The overlap between Puf5 RIP-seq (mRNA only) and a previously reported Puf5 RIP-chip (Gerber et al. 2004). (B). Puf5 RIP-seq enrichment plotted against Puf5 RIP-chip enrichment (Gerber et al. 2004) with the FDR <1% threshold (Hogan et al. 2008); the red trend line represents the least squares regression (N = 171). (C). Overlap between Dhh1 RIP-seq (2 Fold cutoff and 1% FDR) and Dhh1 CLIP-seq targets (2 fold cutoff and 2% FDR) from cells undergoing glucose deprivation stress in the Mitchell et al., 2013 study.

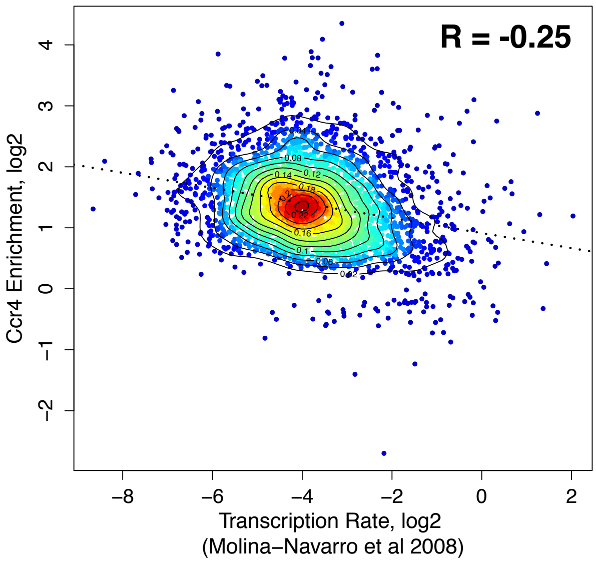

Figure S8: Relationship between Ccr4 recruitment and synthesis.
Ccr4 RIP-seq enrichment was compared to the GRO-chip results from Molina-Navarro et al 2008. The black dashed trend line represents the least squares regression (N = 3133).

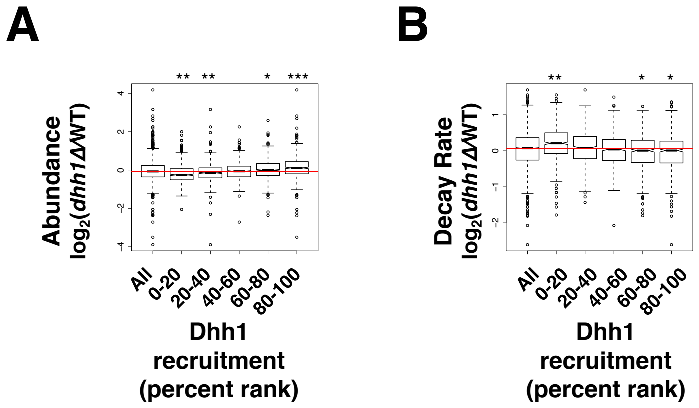

Figure S9: Relationship between Dhh1 recruitment and gene expression changes in a *dhh1Δ* mutant.

(A). Dhh1RIP-seq enrichment values were equally divided into five groups based upon their enrichment percent rank (i.e. 80-100 is the top 20th percentile). Each boxplot represents the log2 change in abundance in a *dhh1Δ* strain relative to a wild-type strain (Sun et al. 2013). Statistical significance was calculated using a Wilcoxon rank sum test with continuity correction. The red line indicates the median of all values. (B). The same as (A) but the boxplots represent the log2 change in decay rates in a *dhh1Δ* strain relative to wild-type. *p-value < 0.001, **p-value < 0.0001, ***p-value < 2.2×10^{-16}.

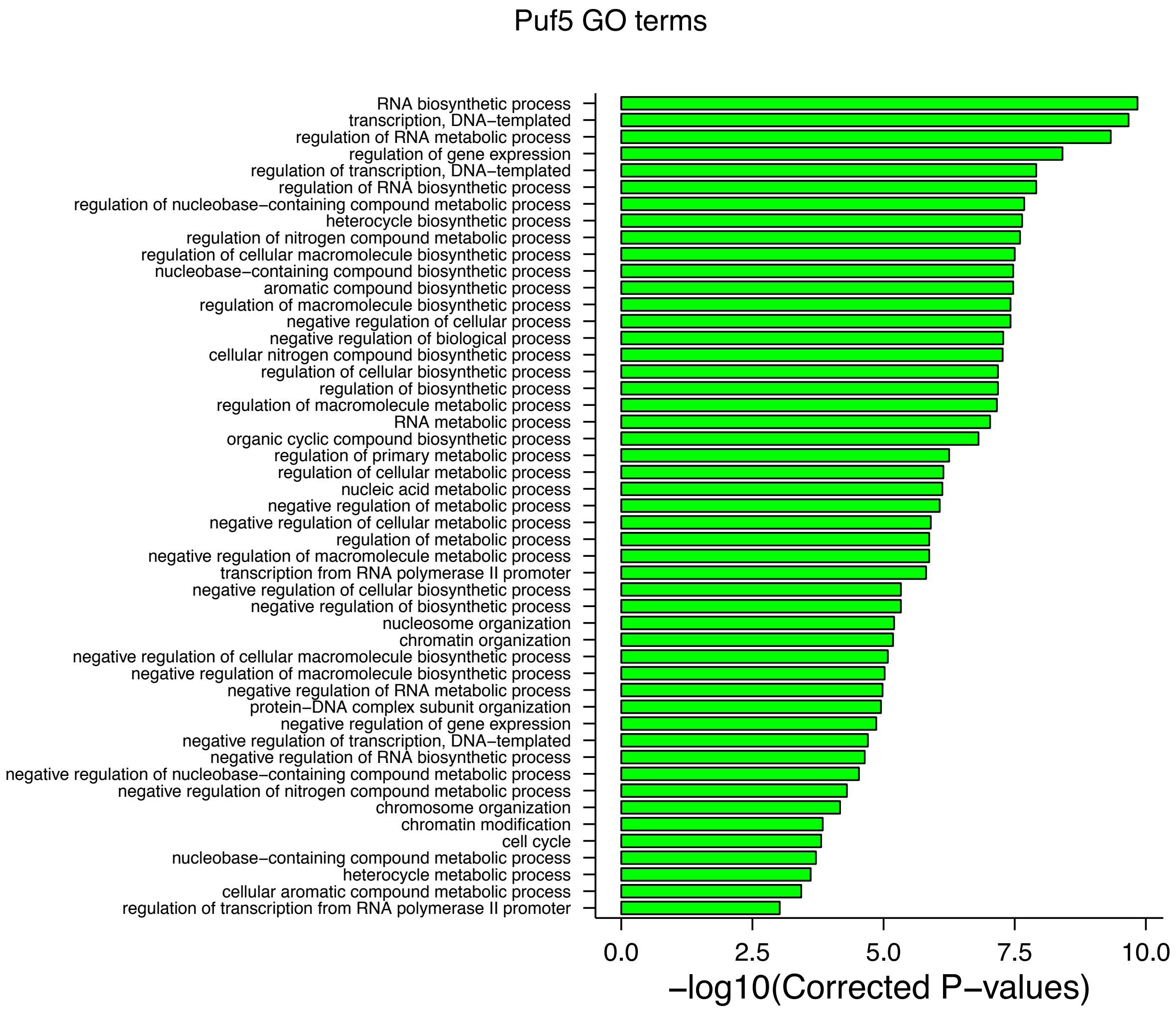

Figure S10: Puf5 GO analysis

GO terms analysis of the top 20th percentile of Puf5 targets (N = 502 genes).

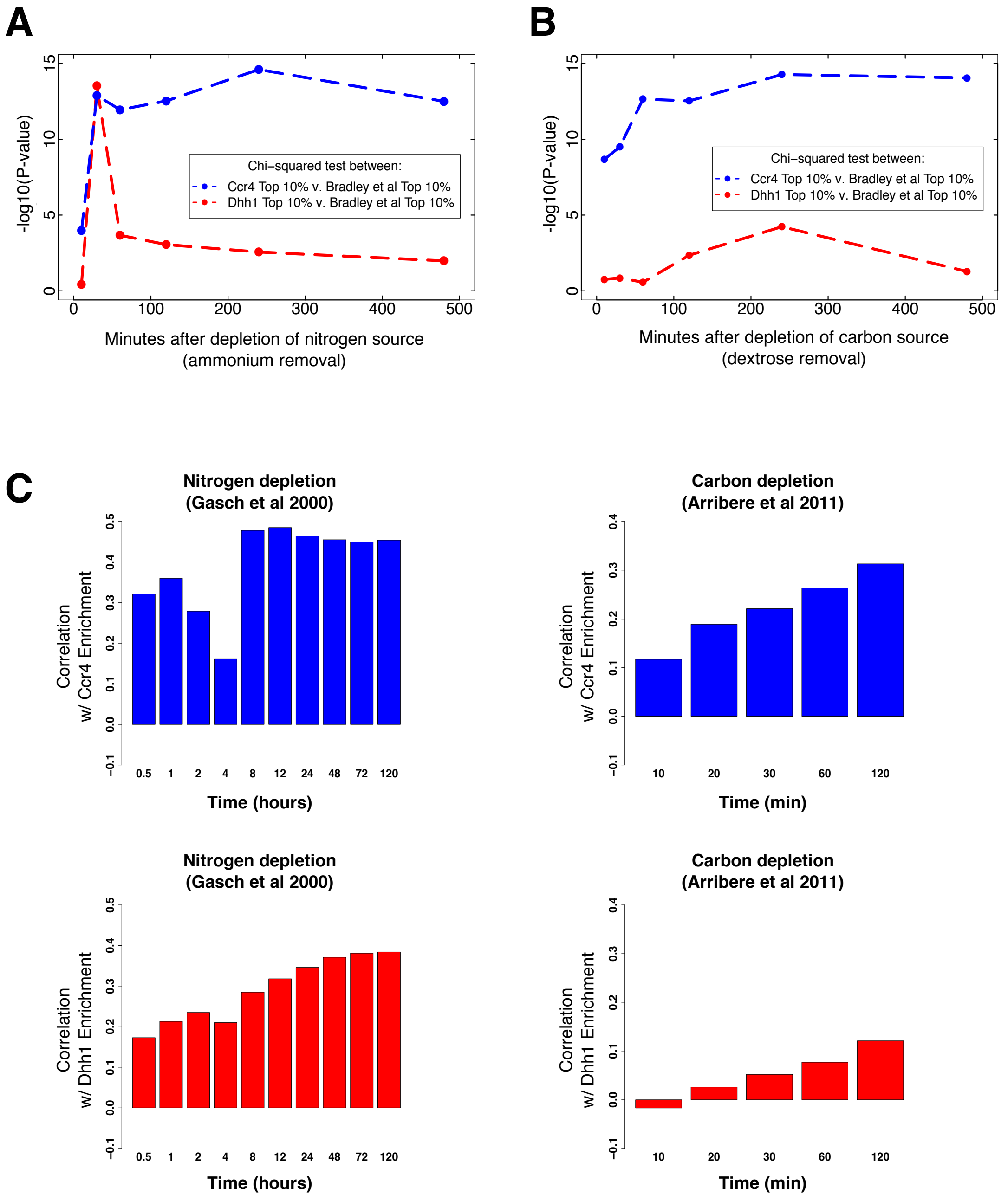

Figure S11: Relationship between recruitment and changes in expression upon nitrogen and carbon starvation

(A)**.** Chi-squared test results comparing genes from the top 10% of Ccr4 (blue) or Dhh1 (red) RIP-seq experiments and genes that were in the top 10% (highly upregulated) after nitrogen depletion (Bradley et al. 2009). (B). The same as (A) except the overlap is displayed between RIP-seq targets and the top 10% of expression changes after carbon source depletion. (C). Pearson correlations were calculated between RIP-seq enrichment and the mRNA expression values from the (Gasch et al. 2000) (nitrogen depletion) and (Arribere et al. 2011) (carbon depletion). Each column represents the correlation of a different time point after the nutrient source was depleted.

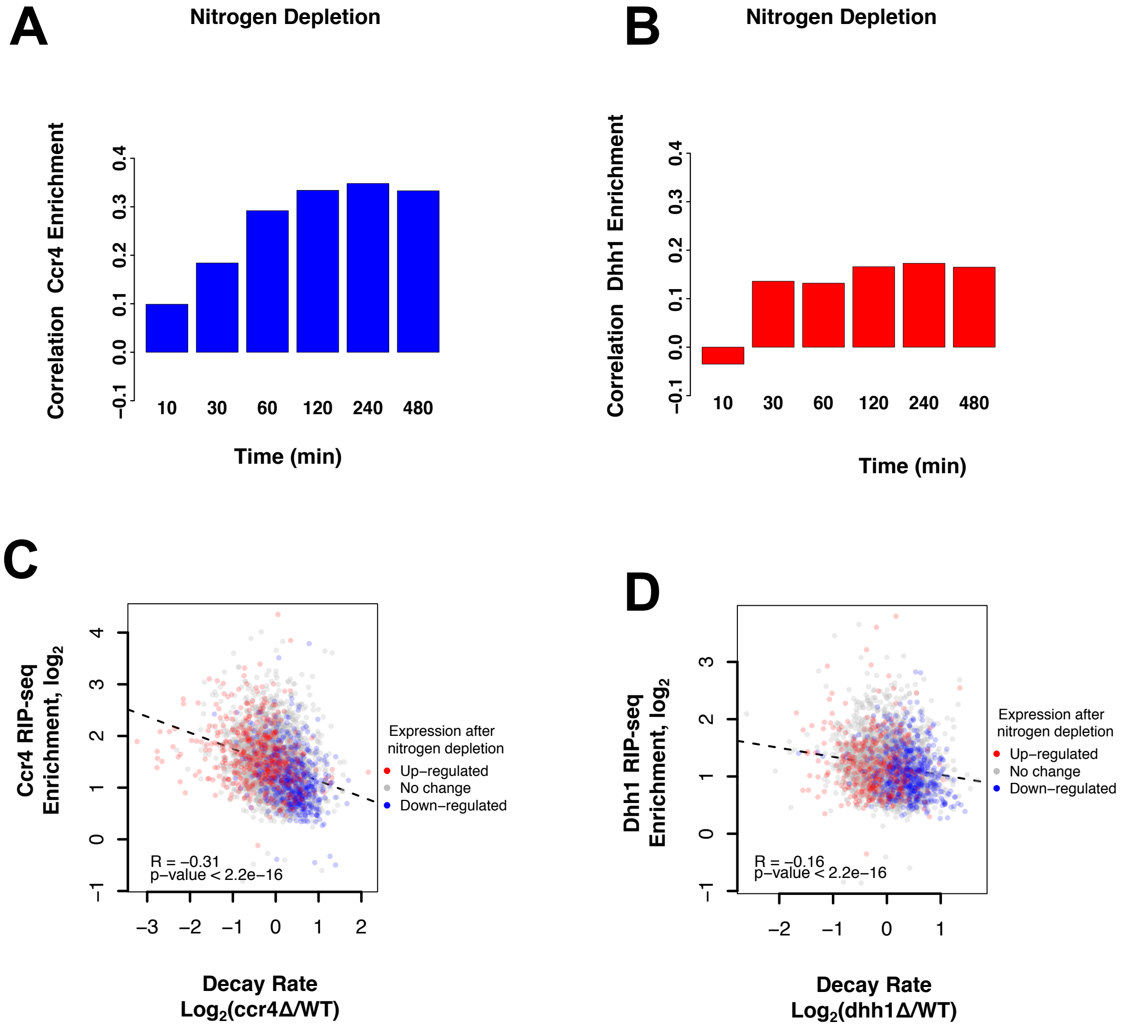

Figure S12. Ccr4 and and Dhh1 recruitment correlates with gene expression changes during Nitrogen starvation. Correlation between Ccr4 (A) and Dhh1 (B) RIP-seq data and expression data from nitrogen depleted cells (Bradley et al. 2009). See legend of Fig 6 for details. Scatter plots comparing RIP-seq enrichment to the change in decay rates in a deletion strain relative to wild-type. The plots for Ccr4 (C) and Dhh1 (D) are shown. The color of data point represents how the expression changed at the 240 min time point after nitrogen depletion. Genes that were down regulated (log2 expression < -1), up-regulated genes (log2 expression > 1) or no-change were colored blue, red and gray, respectively. Scatter plots comparing the recruitment data to changes in decay rates in the mutants described in the x-axis. Genes that change expression at 240 min. time point from Bradley et al. 2009 are colored red (up-regulated), no change (gray) and down-regulated (blue). The Pearson correlation and p-value are included in each plot.

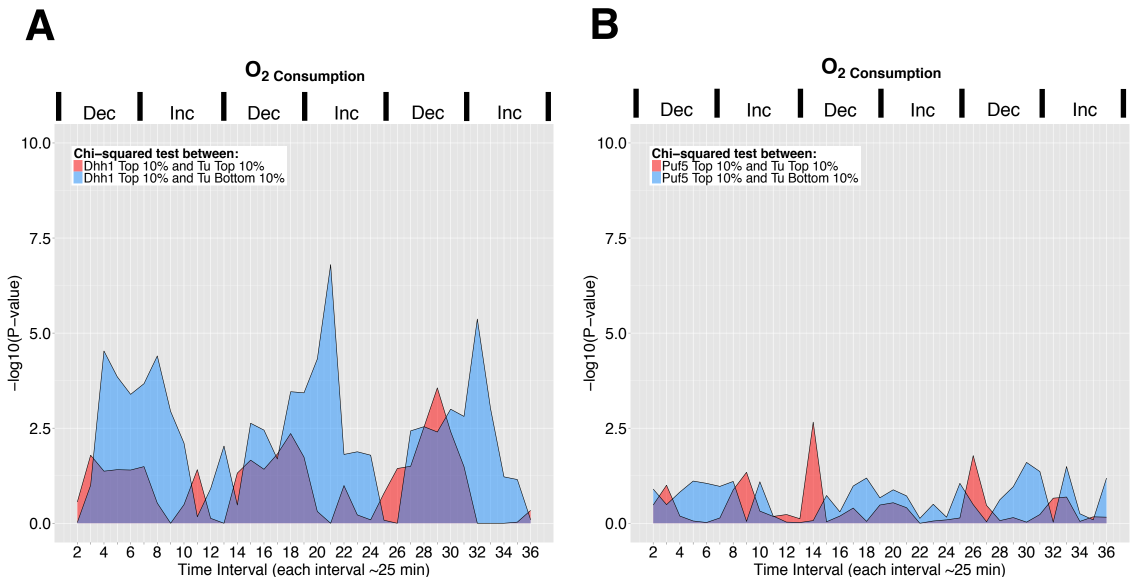

Figure S13: Analysis of Dhh1 and Puf5 RIP-seq enrichment with respect to gene expression during the YMC.
A chi-square test was performed between the Dhh1 (A) and Puf5 (B) highly enriched targets (top 10th percentile) against the up-regulated genes (top 10th percentile of genes (red area)) and down-regulated genes (bottom 10th percentile (blue area)) during the YMC from (Tu et al. 2005). Above the graph represents when oxygen consumption was increasing (Inc) or when it was decreasing (Dec), as determined in (Tu et al. 2005).

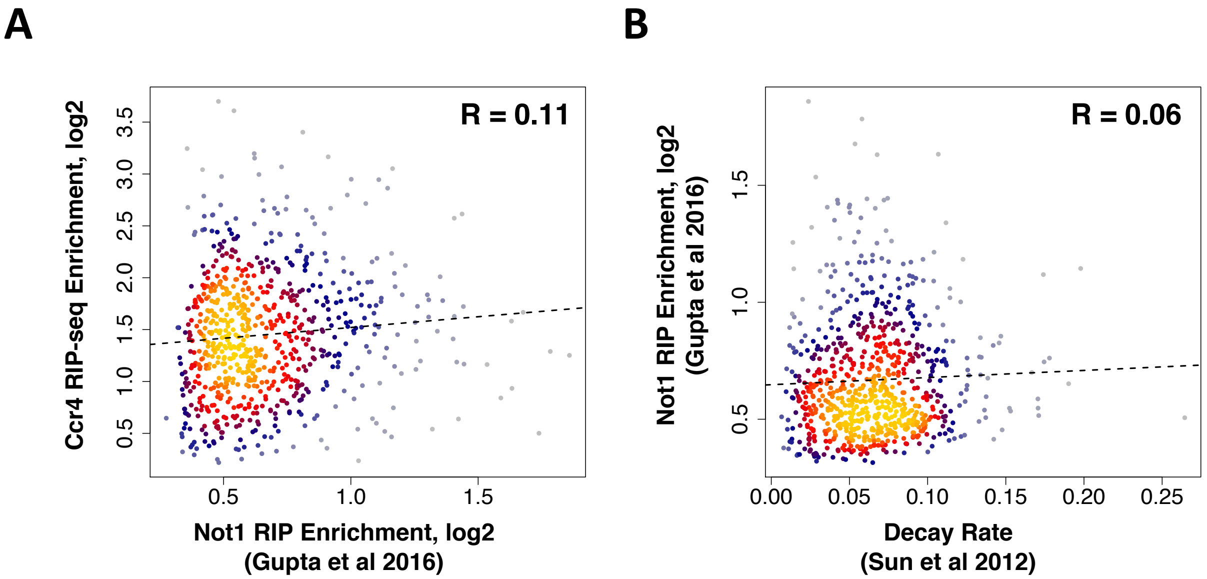

Figure S14: Comparison of Ccr4 and Not1 targets
(A). Ccr4 RIP-seq enrichment plotted against Not1 RIP-seq enrichment from (Gupta et al 2016). (B). Not1 RIP-seq enrichment plotted against the decay rates from Sun et al 2012. The black dashed trend line in both A and B represents the least squares regression. The R values represent Spearman correlations.

A.

1. YPD
2. Glycerol/ethanol
3. YPD → YP wash →YPD 60 min
4. YPD → YP wash →YP 60 min (Glc starvation)
5. SC → NSM wash → SC 4h
6. SC → NSM wash → NSM 4h

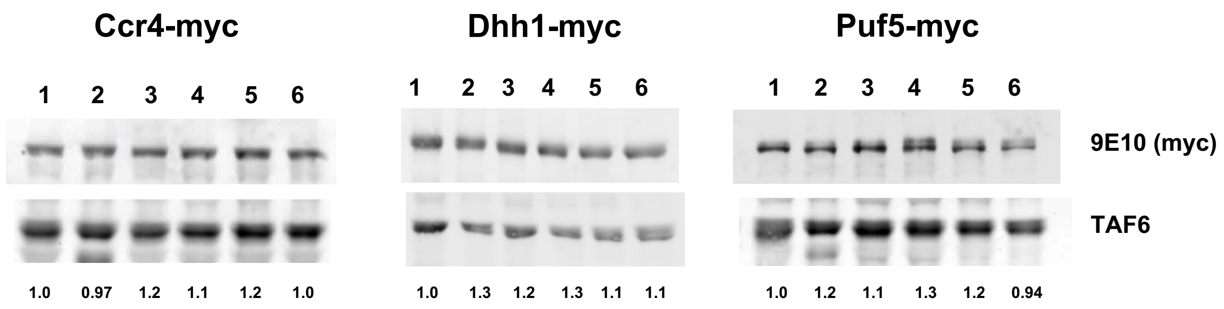

B.

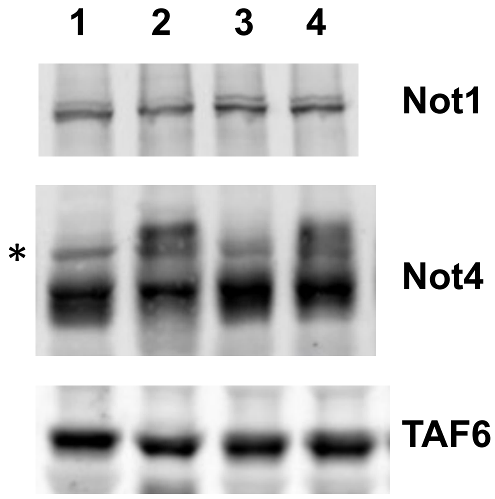

Figure S15: Western blot of proteins under nutrient stress conditions. (A) Ccr4-myc, Dhh1-myc and Puf5-myc were detected using 9E10 under the conditions listed above the panel. TAF6, a component of TFIID and SAGA, was used as a loading control. The numbers below each lane is the quantified signal, corrected for loading, relative to cells grown in YPD rich medium (1). (B). Western blot for Not4 and Not1 under glucose starvation conditions. The growth conditions are indicated above in panel A. The asterisk marks a non-specific band cross reacting with the Not4 antibody.

Table S1: R^2 correlations between replicates illustrates high reproducibility

Replicates	Input	Mock IP	Puf5 IP	Dhh1 IP	Ccr4 IP
1 v. 2	0.979	0.979	0.968	0.983	0.988
1 v. 3	NA	0.939	0.979	0.985	0.937
2 v. 3	NA	0.973	0.982	0.998	0.937

**Table S2: Pearson correlations between RIP-seq enrichment and RNA abundance**			
Study	Puf5	Dhh1	Ccr4
RNA Abundance (Sun et al. 2012)	-0.23	-0.29	-0.41
RPKM (This study)	-0.45	-0.48	-0.50
RPKM (Gerashchenko et al. 2012)	-0.21	-0.29	-0.40

**Table S3: Spearman correlations between RIP-seq Enrichment and gene length**	
Puf5	-0.209
Dhh1	-0.218
Ccr4	-0.045

Table S4: Genes enriched across gene and intron

Ccr4	Dhh1	Puf5
YBL005W-B	YDR500C	YFL031W
YBR090C	YER056C-A	YFL034C-A
YFL031W	YFL034C-A	YGL103W
YFL034C-A	YGL030W	YGL189C
YJR021C	YJL189W	YJL189W
YNL004W	YKL156W	YLR061W
	YML026C	
	YPR153W	

**Table S5: Strains used in this study**	
Strain	Genotype
BY4741	MATa, *his3Δ1*, *leu2Δ0, met15Δ0*, *ura3Δ0*
JR1193	BY4741 with CCR4-13MYC::HIS3
JR1192	BY4741 with DHH1-13MYC::HIS3
JR1462	BY4741 with PUF5-13MYC::HIS3
