# Supplementary material for: Genome-Wide Mapping of Decay Factor–mRNA Interactions in Yeast Identifies Nutrient-Responsive Transcripts as Targets of the Deadenylase Ccr4
Source: G3 (Bethesda). 2017 Nov 20;8(1):315–30. doi: 10.1534/g3.117.300415 (PMC5765359; doi:10.1534/g3.117.300415)
Supplement: Supplementary file 6 [file 315FileS6.docx]

**File S1:** spreadsheet RNA-seq count data

**File S2:** spreadsheet RIP-seq enrichment values

**File S3:** spreadsheet enrichment values at the ends and middle

**File S4:** GO Slim mapping results for Dhh1
